# Blueprints of Architected Materials: A Guide to Metamaterial Design for Tissue Engineering

**DOI:** 10.1002/adma.202408082

**Published:** 2024-10-06

**Authors:** Maria Kalogeropoulou, Anna Kracher, Pierpaolo Fucile, Silvia M. Mihăilă, Lorenzo Moroni

**Affiliations:** ^1^ Complex Tissue Regeneration Department MERLN Institute for Technology‐Inspired Regenerative Medicine Maastricht University Maastricht 6229 ER The Netherlands; ^2^ Division of Pharmacology Department of Pharmaceutical Sciences Utrecht Institute for Pharmaceutical Sciences Utrecht University Universiteitsweg 99 Utrecht 3584 CG The Netherlands

**Keywords:** auxetic metamaterials, meta‐biomaterials, metaimplants, metamaterials, metascaffolds

## Abstract

Mechanical metamaterials are rationally designed structures engineered to exhibit extraordinary properties, often surpassing those of their constituent materials. The geometry of metamaterials’ building blocks, referred to as unit cells, plays an essential role in determining their macroscopic mechanical behavior. Due to their hierarchical design and remarkable properties, metamaterials hold significant potential for tissue engineering; however their implementation in the field remains limited. The major challenge hindering the broader use of metamaterials lies in the complexity of unit cell design and fabrication. To address this gap, a comprehensive guide is presented detailing the design principles of well‐established metamaterials. The essential unit cell geometric parameters and design constraints, as well as their influence on mechanical behavior, are summarized highlighting essential points for effective fabrication. Moreover, the potential integration of artificial intelligence techniques is explored in meta‐biomaterial design for patient‐ and application‐specific design. Furthermore, a comprehensive overview of current applications of mechanical metamaterials is provided in tissue engineering, categorized by tissue type, thereby showcasing the versatility of different designs in matching the mechanical properties of the target tissue. This review aims to provide a valuable resource for tissue engineering researchers and aid in the broader use of metamaterials in the field.

## Graphical abstract

Mechanical metamaterials are architected structures with extraordinary properties, which are defined by their unit cell geometry. Although metamaterials hold significant potential for tissue engineering, their complex design limits their wider use. This review acts as a guide detailing design principles and geometric parameters crucial for metamaterial design, while showcasing applications in tissue engineering, to facilitate metamaterial use in the field.

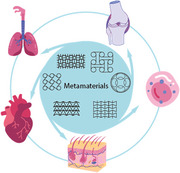

## 1 Introduction

Tissue engineering and regenerative medicine aspire to produce constructs that closely imitate the structure and function of native tissues.^[^ ^1^, ^2^ ^]^ In biological tissues, structure and function are inextricably connected, making the selection of suitable biomaterials and fabrication methods a challenge of significant complexity.^[^ ^3^ ^]^ Seen from a macroscopic perspective, tissues are a functional “whole,” responsible for executing specific tasks. However, from a microscopic point of view, tissues consist of much smaller, repetitive units, connected and assembled via extracellular elements, which usually perform simpler tasks. The shape and organization of those units depend on, but also result in the macroscopic tissue function. Furthermore, biological tissues often exhibit unusual properties that conventional biomaterials can hardly approximate, including negative Poisson's ratios,^[^ ^4^ ^]^ and interfaces between extremely hard and extremely soft materials, such as the osteotendinous junction.^[^ ^5^ ^]^ Recently, a class of rationally designed materials with extraordinary properties, named metamaterials by Walser,^[^ ^6^ ^]^ was introduced as a promising candidate for tissue engineering applications.^[^ ^7^ ^]^

Metamaterials consist of meticulously designed, periodically arranged micro/nano‐units that are responsible for the mechanical properties of the whole material.^[^ ^8^ ^]^ Under loading, the individual building blocks behave like structures, owing to their rational design. When, however, the collective macroscopic behavior of all connected unit cells is considered, it resembles the response of a “new” material, exhibiting distinct mechanical properties.^[^ ^9^ ^]^ Interestingly, the final properties of metamaterials are often superior to those of their constituent materials,^[^ ^10^ ^]^ hence, they provide an attractive template that could be combined with a wide palette of biomaterials as well as fabrication techniques.^[^ ^11^, ^12^ ^]^

Mechanical metamaterials are a subset of metamaterials that exhibit rare or extraordinary elastic mechanical properties, both in the linear elastic and nonlinear region (for large deformations),^[^ ^9^ ^]^ as well as a programmable response to loading.^[^ ^13^ ^]^ Those properties may be zero or negative values of material parameters, such as Poisson's ratio,^[^ ^14^ ^]^ density or compressibility,^[^ ^15^, ^16^ ^]^ as well as ultrahigh strength^[^ ^17^ ^]^ and stiffness. The unit cells of mechanical metamaterials can “deform, rotate, buckle, fold, and snap” under mechanical loading, in a collective manner that yields the desired mechanical properties.^[^ ^10^ ^]^ The design of these fundamental building blocks can be defined by a minimum number of geometric parameters, known as independent parameters, as well as by specific constraints that define the range of values they are allowed to obtain. Changing the values of one or multiple geometric parameters can have a significant effect on the mechanical behavior of the unit cell, and, by extension, of the metamaterial.

A cornerstone in the design and fabrication of unit cells are slender elements, i.e., beams/rods or thin surfaces, connected to each other via hinges or creases.^[^ ^10^ ^]^ One of the most versatile approaches to fabricate such elements is additive manufacturing (AM).^[^ ^7^ ^]^ In tissue engineering, AM has allowed the fabrication of scaffolds with extraordinary complexity and impressive resolution, that can be entirely user‐defined and translated into a printing path by the printing software.^[^ ^18^ ^]^ Taking into consideration the similarity of the hierarchical organization of mechanical metamaterials and biological tissues, it comes as no surprise that the number of published studies on additively manufactured metamaterials for tissue engineering applications shows an ascending trend.^[^ ^19^ ^]^ However, given the advances in available AM methods, the use of mechanical metamaterials in tissue engineering is still relatively limited.^[^ ^14^, ^20^ ^]^ Α possible reason for this delayed dissemination is the design complexity involved in metamaterial fabrication, which goes beyond the conventional grid designs that are available by most AM systems (e.g., the woodpile or honeycomb infill).^[^ ^21^ ^]^

**Figure 1:**
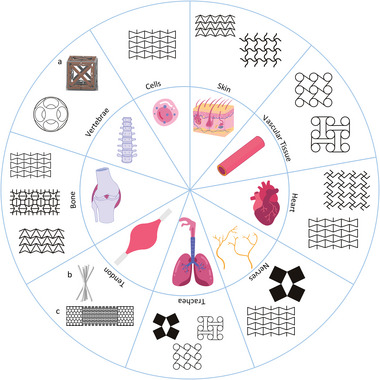
Graphical representation of mechanical metamaterial designs and their applications in different tissues. Created with Canva. (a) Adapted under the terms of the CC‐BY license.^[^ ^22^ ^]^ Copyright 2020, Elsevier Ltd. (b) Reproduced with permission.^[^ ^23^ ^]^ Copyright 2022, Elsevier. (c) Reproduced with permission.^[^ ^24^ ^]^ Copyright 2017, Elsevier.

In this review, we embark on formulating a comprehensive guide for the design of metamaterial constructs for tissue engineering applications. We first present an overview of current metamaterial geometries that have been proposed in the literature for scaffold fabrication. Particular attention has been given to the essential design variables of each presented metamaterial, in order to enhance the reader's understanding of the geometric parameters involved. The effect of the unit cell geometry on the mechanical response of the fabricated construct is also discussed. Additionally, we present the emerging field of artificial intelligence‐aided metamaterial design, which could substantially accelerate metamaterial implementation in biomedical applications. Next, we summarize the state‐of‐the‐art metamaterial applications in tissue engineering. The reviewed studies are organized per tissue type, due to the distinct mechanical requirements of different organs (**Figure** **1**). Finally, we discuss challenges and limitations of the current approaches and offer suggestions for future research. Our aim is to contribute to the investigation and implementation of metamaterial constructs for the engineering of more biomimetic implants and tissues.

## 2 Mechanical Metamaterials: Geometries and Design Parameters

### 2.1 Auxetic Metamaterials

Auxetic metamaterials are among the most widely researched metamaterials.^[^ ^25^, ^26^ ^]^ The word auxetic stems from the Greek word *αυξάνω*, which means to increase. The concept of auxetic metamaterials was first introduced by Lakes in 1986, who prepared triaxially compressed polymeric foams that exhibited a negative Poisson's ratio.^[^ ^27^ ^]^ However, the term was later honed by Evans.^[^ ^28^ ^]^

Poisson's ratio is defined as the negative transverse strain divided by the axial strain in the direction of the stretching force. The Poisson's ratio value (ν) under axial load is calculated using the following formula

(1) $$\nu \;=\;-\frac{\varepsilon_{l}}{\varepsilon_{a}}$$

where $\varepsilon_{l}$ is the lateral strain and $\varepsilon_{a}$ is the axial strain (i.e., along the loading direction).^[^ ^28^ ^]^

The Poisson's ratio of an elastic, isotropic material is connected to the bulk, *B*, and shear modulus *G* via

(2) $$\;\frac{B}{G}=\frac{1}{3}\;\frac{v+1}{0.5-v}$$

In order to ensure that the material is thermodynamically stable, both *B* and *G* need to be positive. The implementation of this non‐negativity to Equation (2) results in the thermodynamically admissible range for isotropic materials of *v* ∈ [− 1, 0.5].^[^ ^29^ ^]^ On the contrary, it has been established that the Poisson's ratios of anisotropic elastic materials have no bounds.^[^ ^30^ ^]^ Poisson's ratio is a continuum concept, meaning that the deformation mechanism of a material can operate at scales ranging from the nano to the macroscale.^[^ ^31^ ^]^ This scale‐independency of Poisson's ratio allows the classification of auxetic materials based on the geometry and deformation of their unit cells, regardless of their scale or fabrication parameters.^[^ ^31^ ^]^

**Figure 2:**
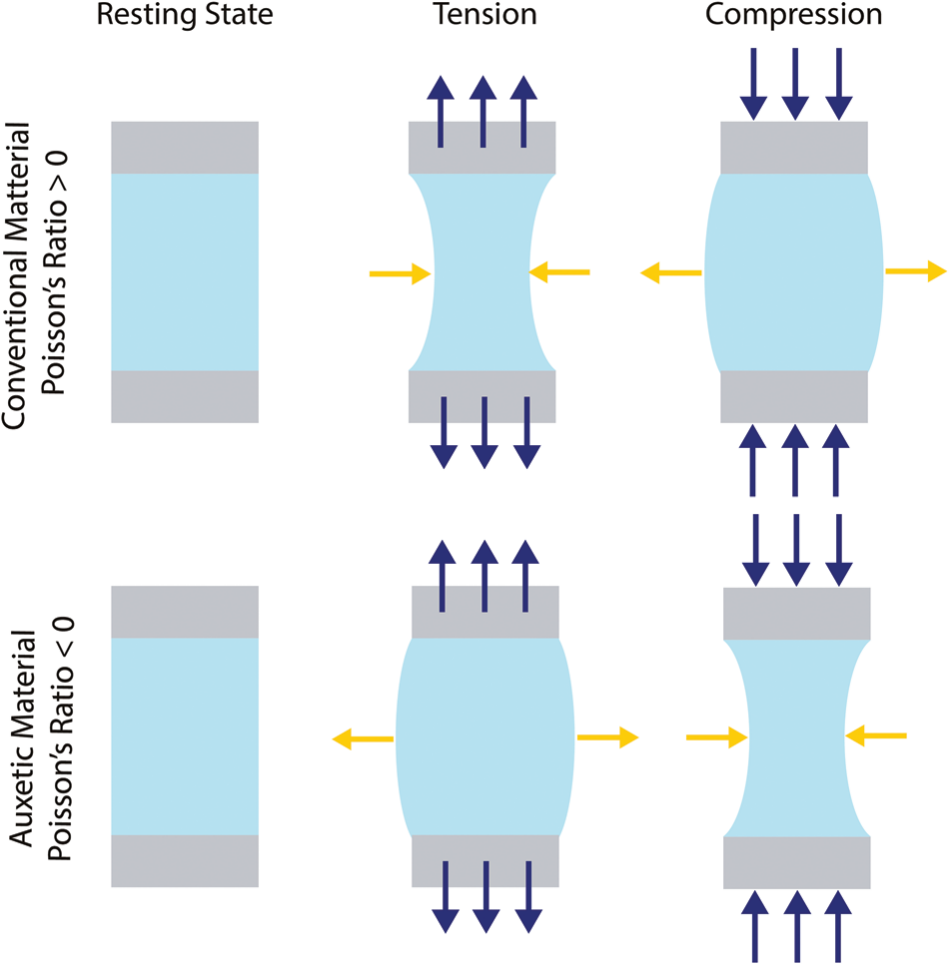
The auxetic effect. Graphical representation of conventional (top) and auxetic (bottom) behavior under tensile and compressive loading. Blue arrows represent the axial force direction. Yellow arrows show the direction of the transverse deformation.

Most conventional materials exhibit positive Poisson's ratio values, meaning that they contract transversely to an applied uniaxial stretch, and expand transversely to uniaxial compression. On the contrary, auxetic materials exhibit the exact opposite behavior, i.e., they expand transversely to an applied stretch and contract transversely in response to compression (**Figure** **2**). Auxetic materials exhibit improved mechanical properties (e.g., higher strength and stiffness) and high energy absorption capacity, regardless of the properties of their constituent material.^[^ ^32^ ^]^ Furthermore, their response to loading makes them attractive for various applications, such as dampening. When auxetic grids are considered, they could be used as patches or casing materials for systems that exhibit complex strain regimes.

Although the auxetic effect is often associated with rationally designed materials, there are reported cases of biological tissues that exhibit a negative Poisson's ratio under specific loading conditions. Williams and Lewis reported negative Poisson's ratios for cancellous bone in the proximal epiphysis of the human tibia.^[^ ^33^ ^]^ Cat^[^ ^34^ ^]^ and cow teat skin,^[^ ^35^ ^]^ axolotl embryonic epithelial tissue,^[^ ^36^ ^]^ cow carotid artery,^[^ ^37^ ^]^ and cow and pig annulus fibrosus of the intervertebral disc^[^ ^38^, ^39^ ^]^ are among the tissues that have been observed to exhibit auxetic behavior. Negative Poisson's ratios are also a characteristic of tendons.^[^ ^4^ ^]^ Interestingly, apart from macroscopic tissues, auxetic behavior has been observed at the microscopic cell scale. Pagliara et al. used atomic force microscopy and reported that the nuclei of mouse embryonic stem cells exhibited an auxetic behavior when transitioning toward differentiation. The auxetic phenotype was, partly, attributed to global chromatin decondensation.^[^ ^40^ ^]^

In the next sections, we summarize the auxetic metamaterial types that have been used in tissue engineering research. More specifically, the independent parameters and the geometric constraints of each unit cell are presented and the design requirements for auxeticity are summarized in each case. Moreover, we also present novel and hybrid designs that have been reported in the literature that could be used as a blueprint for future tissue engineering applications. An overview of both the dependent and independent geometric parameters along with the constraints of each unit cell discussed here is presented in **Table** **1**.

**Table 1:** Overview of the design variables of auxetic unit cells. Light blue lines and corners correspond to the numbered variables. Parameters following a bullet symbol (•) are not depicted in the unit cell design. T* stands for the period of the sinusoidal curve used to design the top and bottom sides of the re‐entrant honeycomb.

Metamaterial	Unit cell	Independent variables		Dependent variables	Constraints	Refs.
Auxetic honeycombs	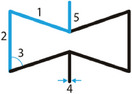	1: *L* 3: *θ* • *α* • *β*		2: *H* = *βL* 4: *t* = *αL* 5: *H*/2	*H* > 2*L*cosθ	[41]
	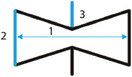	1: *H*_0_ 2: *L*_0_ 3: *L*_1_		• $\alpha =\frac{H_{0}}{L_{1}}$	–	[42]
	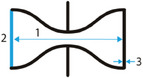	1: *l*_1_ = T* 2: *l*_2_ 3: *t*		• $l_{\sin}=\int_{0}^{\frac{\pi }{2}}\sqrt{1+{\left[\sin{\left(\frac{2\pi }{T}x\right)}^{\prime }\right]}^{2}}dx$	–	[43]
	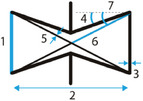	1: *h*_0_ 2: *l*_0_ 3: *t*_0_	4: *θ*_0_ 5: *t*_1_	6: $l_{1}=l_{0}\sqrt{\frac{h_{0}^{2}}{4l_{0}^{2}}+\cos^{2}\theta_{0}}$ 7: $\theta_{1}=\arctan\frac{h_{0}}{2l_{0}\cos\theta_{0}}$	–	[44]
Connected stars	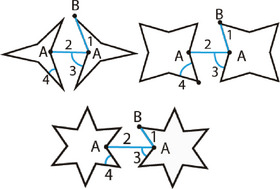	1: AB 2: AA 3: $\hat{AAB}$ 4: $\hat{ABA}$		–	–	[31]
	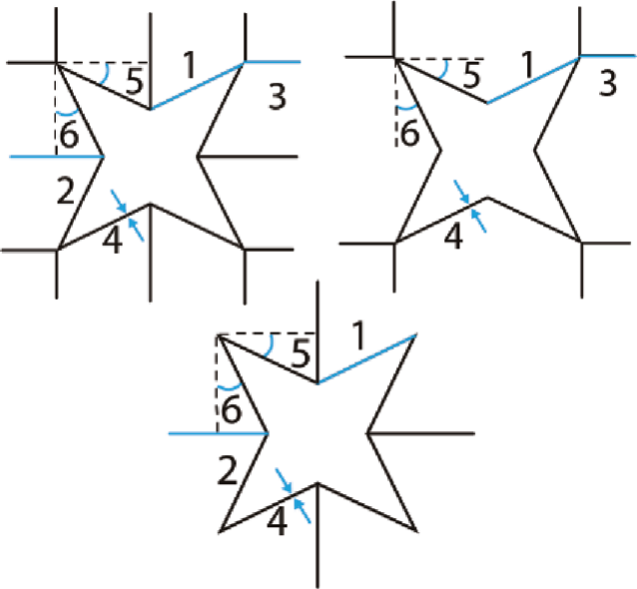	1: *L*_1_ 2: *L*_2_ 3: *L*_3_ 4: *t*	5: *θ*_1_ 6: *θ*_2_ • *w*	• $\alpha =\frac{L_{2}}{L_{1}}\;\;\;\;\;\;\;\;\;\beta =\frac{L_{3}}{L_{1}}$ • $\gamma =\frac{t}{L_{1}}\;\;\;\;\;\;\;\;\delta =\frac{w}{L_{1}}$	sinθ_1_ < αcosθ_2_ αsinθ_2_ < cosθ_1_ θ_1_ + θ_2_ < 90°	[46]
Arrowheads	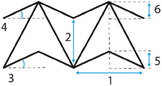	1: *w* 2: *d* 3: *θ*_i_ 4: *θ_i_*_+1_		5: $h_{i}=\frac{w}{2}\tan\theta_{i}$ 6: $h_{i+1}=\frac{w}{2}\tan\theta_{i+1}$	$d-\frac{w}{2}\left(\tan\theta_{i}-\tan\theta_{i+1}\right)>0\;\Leftrightarrow ;$ *d* − *h_i_* + *h*_*i* + 1_ > 0	[48]
	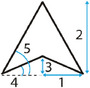	1: *l* 2: *h*_1_ 3: *h*_2_	4: *θ*_1_ 5: *θ*_2_	–	$\frac{h_{1}}{\mathrm{ta}n_{\theta_{1}}}=\frac{h_{2}}{\tan\theta_{2}}=l$	[49]
Missing rib and lozenge	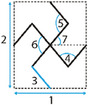	1: *X*_1_ 2: *X*_2_ 3: *α* 4: *θ*_1_	5: *θ*_2_ 6: *ζ* 7: *φ*	–	–	[50]
	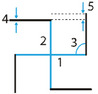	1: *L* 2: *H* 3: *θ*	4: *t* 5: *r*/2	–	–	[52]
Sinusoidal	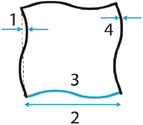	1: *A_n_* 2: *l* • *n* • *t*		• $y=A_{n}\sin\left(\frac{n\pi x}{l}\right)$ 3: $s=\int_{0}^{l}\sqrt{1+{\left(\frac{A_{n}n\pi }{l}\cos\left(\frac{n\pi x}{l}\right)\right)}^{2}}dx$ 4: $w=\frac{tl}{s}$	–	[54]
Chirals	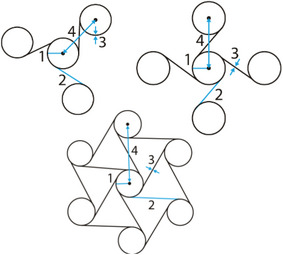	1: *r* 2: *L* 3: *t* 4: *R*		–	–	[59]
Rotating rigids	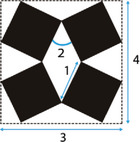	1: *l* 2: *θ*		3,4: $X_{1}=X_{2}=2l\left[\cos\left(\frac{\theta }{2}\right)+\sin\left(\frac{\theta }{2}\right)\right]$	–	[66]
	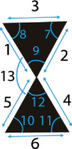	1: *a*_1_ 2: *b*_1_ 3: *c*_1_ 4: *a*_2_ 5: *b*_2_ 6: *c*_2_	7: *α*_1_ 8: *β*_1_ 9: *γ*_1_ 10: *α*_2_ 11: *β*_2_ 12: *γ*_2_	$\left\{ \begin{array}{c} \varphi =\theta -\alpha_{1}+\gamma_{2}=\omega +\beta_{1}-\alpha_{2}\\ \omega =\theta +\gamma_{1}-\beta_{2}=\varphi -\beta_{1}+\alpha_{2}\\ \theta =\omega -\gamma_{1}+\beta_{2}=\varphi +\alpha_{1}-\gamma_{2} \end{array}\right.$ $l_{1}=\sqrt{c_{1}^{2}+c_{2}^{2}+2c_{1}c_{2}\cos\left(\theta -\alpha_{1}-\beta_{2}\right)}$ $l_{2}=\sqrt{b_{1}^{2}+b_{2}^{2}-2b_{1}b_{2}\cos\left(\theta +\gamma_{2}\right)}$ $\alpha_{12}=\cos^{-1}\left(\frac{l_{1}^{2}+l_{2}^{2}-l_{3}^{2}}{2l_{1}l_{2}}\right)$	–	[67]

#### 2.1.1 Re‐entrant Structures

##### Auxetic Honeycombs

**Figure 3:**
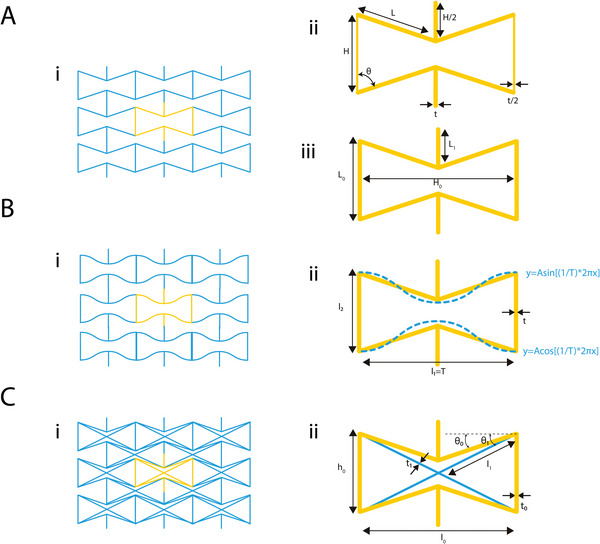
Re‐entrant honeycomb unit cells and variations. A) (i, ii) The typical re‐entrant honeycomb unit cell defined using the vertical and diagonal lengths, *H* and *L*, the re‐entrant angle, *θ*, and the thickness *t*,^[^ ^41^ ^]^ and (iii) the same unit cell defined by the vertical length, *L*_0_, horizontal length, *H*_0_, and connecting rib length, *L*_1_.^[^ ^42^ ^]^ B) Sinusoidal variation of the re‐entrant honeycomb unit cell. The unit cell is defined by the vertical length *l*_2_, the horizontal length *l*_1_ which is equal to the period *T* of the sinusoidal function *y* = *A*sin[(1/*T*)*2π*x*], and the strut thickness *t*.^[^ ^43^ ^]^ C) Composite re‐entrant honeycomb from the combination of rhombic configuration with the re‐entrant honeycomb. The hybrid unit cell is defined by the horizontal length *l*_0_, the vertical length *h*_0_, the diagonal length *l*_1_, the strut thicknesses *t*_0_ and *t*_1_ and the two angles *θ*_0_ and *θ*_1_.^[^ ^44^ ^]^

To model the auxetic foams, Lakes presented an “idealized” re‐entrant unit cell based on hexagonal honeycomb geometries that could be produced by the symmetrical collapse of a 24‐sided polyhedron exhibiting a cubic symmetry.^[^ ^27^ ^]^ Here, re‐entrant is used for describing inward direction or geometries having a negative angle.^[^ ^26^ ^]^ Following Lakes’ model, re‐entrant honeycomb designs became one of the most investigated auxetic geometries (**Figure** **3**).

In terms of unit cell definition, different approaches have been proposed in order to describe the re‐entrant hexagonal unit cell. The unit cell is assumed to have been isolated from a 2D grid of infinite repetitive units. The struts of the hexagonal structure are assumed to have a square cross‐section. In a typical re‐entrant honeycomb lattice, the hexagonal unit can be defined by four independent parameters, namely, *L*,  α,  β,  and θ, where *L* is the inclined strut length, α is the aspect ratio, β is the length ratio, and θ is the re‐entrant angle between the vertical and the inclined struts (Figure 3A(ii)).^[^ ^41^ ^]^ The length of the vertical strut is defined as *H*  =  β*L* while the thickness of the strut cross‐section is given by *t*  =  α*L*. The length of the vertical struts connecting each cell to those above and below is given by *H*/2. Finally, the combination of the above elements can only result in a re‐entrant configuration if the constraint *H* > 2*L*cosθ is satisfied.^[^ ^41^ ^]^

The constituent materials of metamaterials are of little significance regarding the mechanical properties of the final cellular structure. This can be clearly illustrated when considering the formula of the calculation of the relative density of the 2D re‐entrant auxetic structure^[^ ^41^ ^]^

(3) $$\;\rho_{R}=\frac{\left(8+3\beta +2\alpha \right)\alpha^{2}}{{\left(2\sin\theta +2\alpha \right)}^{2}\left(2\beta -2\cos\theta +\alpha \right)}\;$$

It can be deduced from the equation that the relative density of the final grid is defined and can be tuned by the three independent geometric parameters α,  β, and θ. Therefore, careful selection of these parameters may yield constructs with desirable Young's moduli and Poisson's ratios.

The re‐entrant honeycomb unit cell can also be defined without the direct use of the re‐entrant angle, using the vertical length, *H*_0_, the horizontal length, *L*_0_, and the “re‐entrant length”, *L*_1_ (Figure 3A(iii)).^[^ ^42^ ^]^ The aspect ratio of the horizontal to the re‐entrant length is defined as $\;a\;=\frac{H_{0}}{L_{1}}$. A hierarchical design process similar to the formation of fractals was presented, in which the six vertices of the unit cell were replaced with smaller re‐entrant hexagons with the same aspect ratio α. Depending on the iteration of this process, and by extension, the complexity of the resulting construct, the Poisson's ratio could be tuned to different negative values that were smaller than −1.

A variation of the original re‐entrant hexagon was presented by Xu et al. who used a sinusoidal curve to define the unit cell (Figure 3B).^[^ ^43^ ^]^ The design begins from the sinusoidal function $\;y\;=\;A\sin(\frac{1}{T}2\pi x)$, where *A* is the amplitude and *T* the period. By isolating a single period of the sinusoidal function from one local maximum to the next, and connecting the first maximum with the minimum and, subsequently, the minimum with the next maximum, one can derive the top side of the re‐entrant hexagon. The bottom side of the unit cell can be derived from the same procedure with a phase shift of π/2 (or, equivalently, by using the cosine function). Therefore, the horizontal length of the unit cell is equal to the period of the sinusoidal curve, i.e., *l*_1_ =  *T*. The vertical strut length is arbitrarily defined as *l*_2_ and the in‐plane thickness of the struts is given by *t*. Although this model does not provide general equations that relate the independent geometric parameters with each other, it is still of interest as it introduces a new approach for extracting re‐entrant honeycombs that could be fabricated using continuous sinusoidal curves instead of polylines. The relative density of the honeycomb in this case is given by

(4) $$\rho \;=\frac{A_{s}}{A_{\mathrm{total}}}\;=\sum_{i=1}^{N}\frac{L_{i}t}{L_{1}L_{2}}=\frac{\left(256l_{\sin}+248l_{2}\right)t}{L_{1}L_{2}}\;$$

where *A*_s_ is the area of the solid part of the unit cell, *A*_total_ is the total cross‐sectional area of the cell, *L_i_* is the total length of the *i*th single cell, *t* is the thickness of the cell wall, $l_{\sin}=\int_{0}^{\frac{\pi }{2}}\sqrt{1+{\left[\sin{\left(\frac{2\pi }{T}x\right)}^{\prime }\right]}^{2}}dx\;$ is the one cycle length of the curve, *l*_2_ is the straight arm length of the single cell, *L*_1_ and *L*_2_ are the length and width of the honeycomb structure sample. A thorough study of sinusoidal honeycombs can be found in the work of Shankar et al.^[^ ^45^ ^]^

A composite honeycomb design was presented by Fu et al. from the combination of a rhombic configuration and the conventional re‐entrant honeycomb (Figure 3C).^[^ ^44^ ^]^ In this approach, the unit cell is constructed by the conventional hexagonal cell reinforced with four additional struts. Similarly to the definitions presented above, the re‐entrant hexagons are defined by their vertical length *h*_0_, the length of the inclined struts, *l*_0_, and their thickness *t*_0_. The thickness of the reinforcing walls is defined as *t*_1_ and their equal lengths are given by *l*_1_. The inclined walls of the re‐entrant hexagon form an angle θ_0_ with the horizontal direction, while the angle between the reinforcing walls and the horizontal direction is given by θ_1_. The independent parameters of the representative block of the new honeycomb are related with the following formulas

(5) $$\;l_{1}=l_{0}\;\sqrt{\frac{h_{0}^{2}}{4l_{0}^{2}}+\cos^{2}\theta_{0}}$$

(6) $$\;\theta_{1}=\arctan\frac{h_{0}}{2l_{0}\cos\theta_{0}}\;$$

The deformation of honeycomb lattices is attributed to the presence of the vertical rib of the unit cell, which results in changes of the re‐entrant angle depending on the mode of axial loading (compression or tension). By making adjustments to the design of the original unit cells, the mechanical properties of the final construct can be tailored to match the properties of various tissues.

Re‐entrant honeycombs and their variations are among the most popular metamaterials in the existing tissue engineering applications, perhaps due to their simpler design and homogeneous pore size.

##### Connected Stars

**Figure 4:**
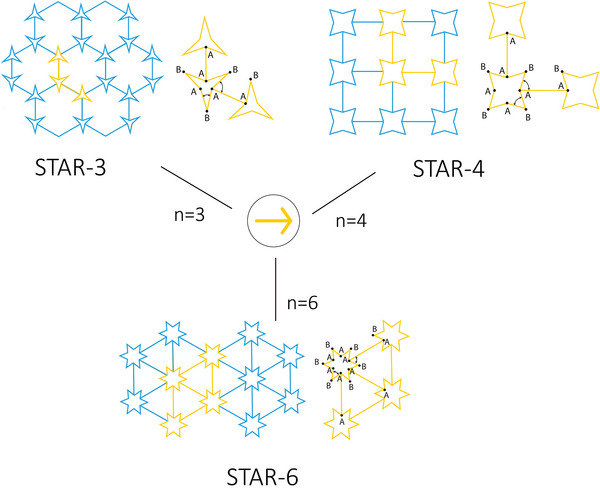
Lattice configurations and unit cells of connected stars. The arrow‐shaped building block of the stars is presented in the middle. The resulting stars consist of 3 (STAR‐3), 4 (STAR‐4) or 6 (STAR‐6) arrow blocks. The points A and B in each geometry indicate the inner and outer points of each star‐shaped unit cell.^[^ ^31^ ^]^

Inspired by the hexagonal re‐entrant honeycomb, Grima et al. suggested that this geometry can be built using “arrow‐shaped building blocks,” with a rotational symmetry of *n* = 1.^[^ ^31^ ^]^ In fact, according to the authors, it is this arrow‐shaped unit that is responsible for the auxetic behavior of the final structure. By increasing the rotational symmetry factor to *n* = 3, 4, and 6 they presented novel structures, namely, STAR‐3, STAR‐4, and STAR‐6, which also exhibit auxetic properties (**Figure** **4**).

**Figure 5:**
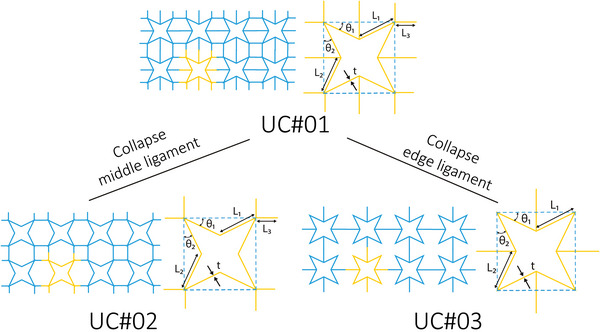
Connected star lattices designed by addition or subtraction of connecting ligaments. Starting from the unit cell UC#01, removing the middle horizontal ligament results in UC#02 while removal of the edge ligament yields UC#03.^[^ ^46^ ^]^ The characteristic lengths, *L*_1_, *L*_2_, *L*_3_, the strut thickness, *t*, and the angles *θ*_1_, *θ*_2_ are indicated in each unit cell design.

The geometric variables that need to be considered when designing star grids are the bond length *AB*, which refers to the dimension of the stars, and the bond length *AA*, which represents the length of the elements that connect neighboring stars. Additionally, two angles need to be defined, namely $\hat{ABA}$, which is the acute inner angle of the star, and $\hat{AAB}$, which is equivalent to the angle θ of the re‐entrant honeycomb unit cell. Finally, the torsion angles $\hat{\varphi AB\;}$ and $\hat{\varphi AA\;}$ (not depicted) between four connected atoms A–A–B–A and B–A–A–B must be set to ±180° and 0°/±180°, respectively, in order to constrain the system to remain planar. The values of the torsion angles could potentially be changed when nonplanar structures are considered. Ai and Gao presented three types of star‐based re‐entrant periodic lattice structures, UC#01, UC#02, and UC#3 (**Figure** **5**).^[^ ^46^ ^]^ All three proposed unit cells are based on the STAR‐4 geometry^[^ ^31^ ^]^ and differ in the number of connections with their neighboring stars. The unit cell UC#01 can be fully defined by seven independent geometric parameters, namely, *L*_1_,*L*_2_, *L*_3_, θ_1_, θ_2_, *t*, and w, where *L*_1_,*L*_2_, *L*_3_ are the different strut lengths, θ_1_, θ_2_ are the re‐entrant angles, *t* is the thickness of the struts and *w* the out‐of‐plane width of the unit cell.^[^ ^46^ ^]^ Upon defining the independent parameters, four new nondimensional parameters can be introduced

(7) $$\alpha \;=\frac{L_{2}}{L_{1}}\;,\beta \;=\frac{L_{3}}{L_{1}}\;,\gamma \;=\frac{t}{L_{1}}\;,\delta \;=\frac{w}{L_{1}}\;$$

where α is the re‐entrant strut ratio, β is the external connection length ratio, γ is the slenderness ratio, and δ is the aspect ratio. To prevent the contact or intersection of the struts, the following constraints should be satisfied

(8)

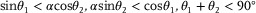




The other two unit cells can be designed by reducing the connections of UC#01 with the neighboring stars. More precisely, in UC#02 only the edge vertices are connected, whereas in UC#03 only the middle vertices are used as connection points. In the special case where *L*_1_ = *L*_2_ and θ_1_ = θ_2_ , all the unit cells will exhibit isotropic symmetry on the plane defined by two connected struts. Similarly to the re‐entrant hexagons, the relative densities of the final metamaterials are fully dependent, and therefore can be tuned by the geometric parameters of the unit cells^[^ ^46^ ^]^

(9) $$\;\rho_{\mathrm{UC}\#01}=\frac{\left(1+a+3\beta +0.5\sin\theta_{1}+0.5\alpha \sin\theta_{2}\right)\gamma }{\left(\beta +\cos\theta_{1}\right)\left(\beta +a\cos\theta_{2}\right)}\;$$

(10) $$\;\rho_{\mathrm{UC}\#02}=\frac{\left(1+a+2\beta \right)\gamma }{\left(\beta +\cos\theta_{1}\right)\left(\beta +a\cos\theta_{2}\right)}\;$$

(11) $$\;\rho_{\mathrm{UC}\#03}=\frac{\left(1+a+\beta +0.5\sin\theta_{1}+0.5\alpha \sin\theta_{2}\right)\gamma }{\left(\beta +\cos\theta_{1}\right)\left(\beta +a\cos\theta_{2}\right)}\;$$

The auxetic response of the interconnected‐star geometries is based on the “opening” and “closing” of the star nodes, which, depending on the applied strain, may result in large changes in the pore size. This property should be taken into account when considering tissue engineering applications as tissue growth might progressively limit the freedom of motion of the star ribs.

##### Arrowheads

**Figure 6:**
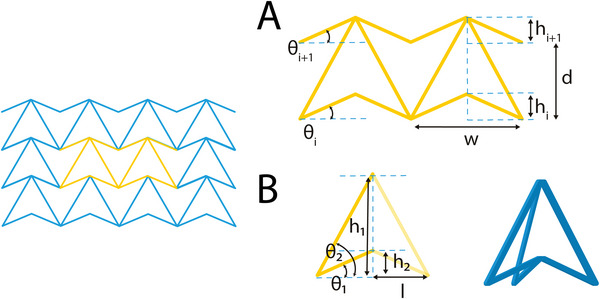
Arrowhead lattice and unit cells. A) Arrowhead unit cell design based on the vertical lengths, *h_i_*, *h_i_*_+1_, *d*, the horizontal length *w*, and the angles *θ_i_* and *θ_i_*_+1_.^[^ ^48^ ^]^ B) Arrowhead unit cell based on the vertical symmetry definition, using the heights *h*_1_, *h*_2_ and the angles *θ*_1_ and *θ*_2_.^[^ ^49^ ^]^

Arrowhead‐like unit cells were originally presented by Larsen et al. as the result of numerical topology optimization for compliant structures.^[^ ^47^ ^]^ Later, the arrowhead geometry was connected to the original hexagonal re‐entrant honeycomb, by modifying the re‐entrant elements from hexagons to triangles, hence the arrowhead resemblance of the final lattice. To define the unit cell of the arrowhead lattice, four independent geometric parameters are needed: the lengths *w* and *d*, and the angles θ_*i*_ and θ_*i* + 1_ (**Figure** **6**A).^[^ ^48^ ^]^ Two more parameters, the vertical distances *h_i_* and *h*_*i* + 1_ can be defined based on the two angles, as: $h_{i}=\frac{w}{2}\;\tan\theta_{i}$ and $h_{i+1}=\frac{w}{2}\;\tan\theta_{i+1}$. To avoid contact or crossing of the struts, the following constraint is to be satisfied

(12) $$d-\frac{w}{2}\left(\tan\theta_{i}-\tan\theta_{i+1}\right)>0\;\Leftrightarrow ;\;d-h_{i}+h_{i+1}>0$$

In a recent study by Guo et al., the unit cell of the arrowhead auxetic lattice was designed by defining the symmetrical half‐arrowhead, and subsequently mirroring it along its axis of symmetry (Figure 6B).^[^ ^49^ ^]^ In this approach, five independent geometric parameters are required for the unit cell definition, the half width of the arrow, *l*, the top and bottom arrowhead heights, *h*_1_ and *h*_2_, and the angles between the horizontal line and the inclined struts of the unit cell θ_1_ and θ_2_. For the proper generation of the arrow‐head structure, the following conditions are to be satisfied

(13) $$\;\frac{h_{1}}{\mathrm{ta}n_{\theta_{1}}}=\frac{h_{2}}{\tan\theta_{2}}\;=\;l$$

The resulting unit cell can be used to generate both 2D auxetic grids as well as 3D constructs. In order to obtain a 3D building block, the polygon tessellation approach has been proposed. Briefly, the defined half‐arrow is rotated around its axis of symmetry until its lower vertex coincides with the vertex of an isosceles triangle, a square or a hexagon. Based on this principle, three unit cells were defined, resulting in 3D triangle, square, and hexagon tessellated trusses.^[^ ^49^ ^]^ For the complete definition of each of the 3D unit cells, additional parameters including the side length of the tessellated base‐shapes as well as the thickness of the inclined sides should also be defined.

The ease of expansion of this unit cell to a 3D configuration makes it an attractive choice for tissue engineering applications focusing on larger scaffolds, e.g., for bone tissue engineering. Additionally, the freedom of designing 3D arrowhead unit cells form a tessellated space could be further explored by combining different base geometries in the same construct, in order to provide scaffolds and implants that could best fit the affected site.

##### Square and Lozenge Grids

**Figure 7:**
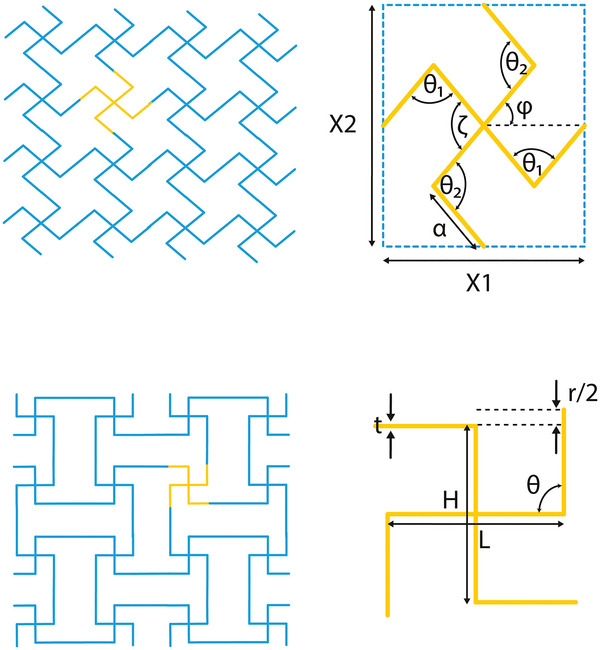
Missing rib and square grid lattices and unit cells. A) Missing rib grid and unit cell, based on the lengths *X*_1_, *X*_2_, *α*, and angles *θ*_1_, *θ*_2_, *ζ*, *φ*.^[^ ^50^ ^]^ B) Square grid design and unit cell, using the lengths *Η*, *L*, and *r*/2, the thickness, *t*, and the angle *θ*.^[^ ^52^ ^]^

In an effort to better explain the negative Poisson's ratio exhibited by reticulated foams, Smith et al. introduced a model based on the missing‐rib principle (**Figure** **7**).^[^ ^50^ ^]^ The rationale behind this approach was based on the observation that during foam compression, cell ribs would fracture, changing the properties of the material from conventional to auxetic. Starting from a typical square grid and removing or “breaking” certain ribs, the square grid unit cell was obtained. The independent geometric parameters needed for this design are the width and height of the circumscribed rectangle, *X*1 and *X*2, respectively, the length of the ribs, *a*, and the four angles θ_1_, θ_2_, ζ, and φ. It has been established that if the structure is based on a square unit cell, the Poisson's ratio of the final construct will be −1.^[^ ^50^, ^51^ ^]^ The auxetic square grid unit cell has also been designed based on the horizontal and vertical lengths, *H* and *L*, respectively, the flap angle, θ, formed between them, the strut thickness, *t*, and the adjoining length between neighboring unit cells, *r*.^[^ ^52^ ^]^ Based on the above geometric parameters, the auxetic response of the square grid could yield Poisson's ratios as low as −7, by altering the *H*/*L* ratio and flap angle θ.^[^ ^52^ ^]^ Computational predictions indicated Poisson's ratios as low as −20, however this value was not experimentally validated due to buckling limitations.

Lozenge grids are very similar to square grids, with their major difference being the length of the ribs connecting adjacent unit cells. Increasing the rib length has been reported to contribute to the rotation of the unit cell upon loading, which is a response more similar to that of chiral structures (see Section 2.1.3).^[^ ^53^ ^]^

Missing‐rib geometries offer homogeneous pore size across the entire lattice and could be easily tailored to match the desired porosity values for specific tissue applications, while following the geometric requirements for auxeticity. However, it should be noted that the sharp angles present in the unit cell could result in stress concentrations during loading, thus limiting the strain range where the construct exhibits the target negative Poisson's ratio. Therefore, a fine‐tuning of the unit cell angles with the expected strain range and rate of the target tissues should be performed (Figure 7).

##### Sinusoidal Ligaments

**Figure 8:**
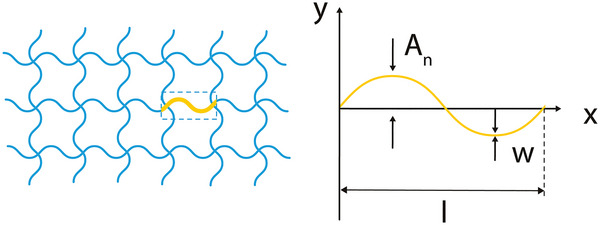
Sinusoidal ligament‐based lattice and unit cell with the essential design parameters of the waveform: amplitude *A_n_*, thickness, *w*, and length, *l*.^[^ ^54^ ^]^

As their name indicates, sinusoidal ligaments are a class of auxetic, re‐entrant lattices that are constructed based on the sinusoidal curve.^[^ ^43^, ^45^ ^]^ Straight edges in conventional square lattices can be replaced with sinusoidal ligaments, hence, transforming a conventional geometry to an auxetic structure (**Figure** **8**).^[^ ^54^ ^]^ The sinusoidal ligaments are described by the typical sinusoidal equation

(14) $$y\;=A_{n}\;\sin\left(\frac{n\pi x}{l}\right)$$

where *A_n_* is the amplitude, *n* is the number of half wavelengths, and *l* is the length of the straight beam that is being replaced. Here, we consider that the lattice is laying on the *xy* plane. The length of the sinusoidal ligament is given by the formula

(15) $$s\;=\int_{0}^{l}\sqrt{1+{\left(\frac{A_{n}n\pi }{l}\cos\left(\frac{n\pi x}{l}\right)\right)}^{2}}\;dx$$

According to the mass equivalent assumption, the width of the new ligaments is given by

(16) $$w\;=\frac{tl}{s}\;$$

where *t* is the thickness of the original ligament. An additional fourth geometric parameter, *b*, has also been proposed in the literature to account for the out‐of‐plane thickness of the sinusoidal lattice, which is important when additive manufacturing approaches are considered.^[^ ^55^ ^]^

Apart from the typical square grid, hexagonal, triangular, and kagome lattices have been fabricated using sinusoidal ligaments, demonstrating the potential to use this approach for transforming a host of conventional topologies.^[^ ^54^ ^]^ Moreover, by introducing a phase shift between the ligaments of the lattice with various combinations, more auxetic structures can be obtained. It has been reported that these arrangement as well as the number of ligaments used in the lattice design can have a significant effect on the final (meta)material behavior, yielding lattices that could withstand large deformations, with Poisson's ratios both in the positive and the negative regime.^[^ ^56^ ^]^ In the case of volumetric approaches, the 3D form of the unit cell of the sinusoidal ligaments lattice has been designed as the 13th eigenmode of a regular cubic unit cell and characterized extensively by Warmuth et al.^[^ ^57^ ^]^

Apart from the auxetic effect, the curved sinusoidal ligaments could aid in local stress alleviation during loading, which could, in turn, increase the strain range of the auxetic effect. This feature could be further explored for tissues subject to large deformations, such as the myocardium.^[^ ^58^ ^]^ Furthermore, the smooth sinusoidal curves provide an attractive design approach for tubular constructs (e.g., vascular stents), as the lack of sharp angles minimizes the risk of out‐of‐plane sharp elements that could injure the surrounding tissue.

#### 2.1.2 Chiral Structures

**Figure 9:**
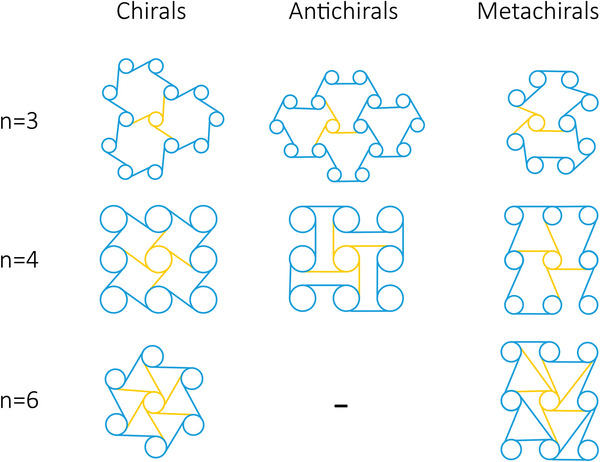
Chiral, antichiral, and metachiral configurations with 3 (trichiral), 4 (tetrachiral), and 6 (hexachiral) tangent ribs on the central node of the unit cell.^[^ ^59^ ^]^

Chiral unit cells are composed of a central node with tangentially attached ligaments (typically, 3, 4, or 6, commonly known as tri‐, tetra‐, and hexachiral geometries).^[^ ^59^, ^60^ ^]^ The ligaments are arranged in such a way that the unit cell exhibits rotational symmetry of order three, four or six, respectively (**Figure** **9**). This type of connectivity makes the unit cell “chiral,” meaning that it may be designed as right‐handed or left‐handed. The two versions of the basic geometry are mirror‐images of each other; hence, they cannot be superimposed. Moreover, the resulting lattice is constructed only by unit cells demonstrating the same chirality and is also chiral.^[^ ^59^ ^]^ The central node geometry is usually circular. However, alternative closed loop topologies have been presented in the literature.^[^ ^61^, ^62^, ^63^ ^]^

When a uniaxial stress is applied to the chiral unit cell, a torque is generated, which results in the bending of the tangential ligaments into a sigmoid shape and the subsequent rotation of the central nodes. Since the ligaments remain attached to the node, the deformation of chiral structures corresponds to a change in area without a change of shape.^[^ ^64^ ^]^ Negative Poisson's ratio values have been reported for chiral lattices under various loading schemes.^[^ ^60^, ^63^, ^64^ ^]^

Attaching adjacent nodes on the same side of the connecting ligaments results in a configuration commonly known as “antichiral.” In other words, the nearest‐neighboring nodes of an antichiral structure exhibit opposite chirality.^[^ ^60^, ^65^ ^]^ Compared to their chiral counterparts, anti‐trichiral and anti‐tetrachiral lattices have been reported to exhibit lower Young's moduli, and could potentially be considered for targeting softer tissues than chiral geometries.^[^ ^60^ ^]^

In both chiral and antichiral patterns, the geometric parameters that affect the mechanical properties of the final structures are the radius of the nodes, *r*, the length of the ligaments, *L*, the cell wall thickness *t* and the distance between the centers of adjacent nodes *R*. The ratio *R*/*r* can be used to control the porosity of the final lattice.^[^ ^64^ ^]^ It is worth mentioning that other parameters could be considered when the central node is not a cylindrical element. In the general anti‐tetrachiral model presented by Gatt et al., the length and thickness values of ligaments on the horizontal and vertical direction were independently considered, showcasing an additional set of parameters that could be used for programming the mechanical response of the final metamaterial.^[^ ^65^ ^]^

By relaxing the constraint that a chiral unit cell should exhibit a rotational symmetry of order *n*, where *n* is the number of ligaments attached to its node, it becomes possible to construct other topologies which are known as “metachirals.”^[^ ^59^ ^]^ Using rectangular nodes, a Poisson's ratio lower than −1 can be achieved, owing to the high anisotropy of the system.^[^ ^59^ ^]^ Hence, by relaxing the constraints inherent to the chiral and antichiral definitions, more metamaterial geometries could be generated. Chiral structures can even further be combined with patterns from other classes of auxetic metamaterials. For instance, a novel metamaterial was formulated by incorporating basic chiral structures with re‐entrant hexagonal honeycomb central nodes.^[^ ^61^ ^]^

Due to the inherent rotation of their nodes under loading, chiral and metachiral‐based constructs could be used for the delivery of multiple mechanical stimulation types to cells in vitro, as they can combine linear with torsional deformations. The existence of two different pore geometries in this lattice design is a point that could require some attention for tissue engineering applications, in case homogeneous porosity is a prerequisite of the target organ. Moreover, the rotation feature of chiral metamaterials makes them attractive choices for self‐expanding devices, such as stents.

#### 2.1.3 Rotating Rigid Structures

**Figure 10:**
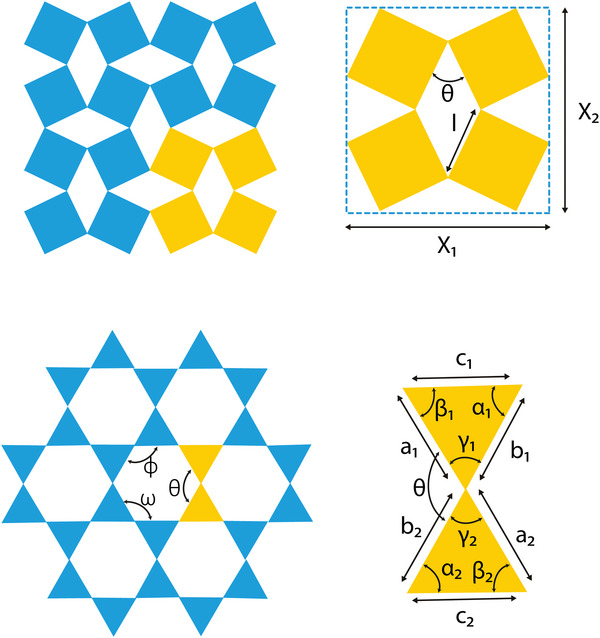
Rotating rigid unit cells and design parameters. A) Rotating squares. The unit cell is designed using the lengths *X*_1_, *X*_2_, and *l* and the angle *θ*.^[^ ^66^ ^]^ B) Rotating triangles. The unit cell is designed using the lengths *a*_1_, *a*_2_, *b*_1_, *b*_2_, *c*_1_, *c*_2_, and the angles *α*_1_, *α*_2_, *β*_1_, *β*_2_, *γ*_1_, *γ*_2_.^[^ ^67^ ^]^

Auxetic metamaterials based on rotating rigid structures can be built up from different shapes connected together at their vertices by hinges (**Figure** **10**). The concept of rotating rigid models was originally reported by Grima and Evans when they reported the auxetic behavior of rotating squares.^[^ ^66^ ^]^ By using the principle of energy conservation, they showed that the idealized rotating square system will always maintain its aspect ratio and will, thus, exhibit constant Poisson's ratios of −1.^[^ ^66^ ^]^ The unit cell of the rotating squares structure consists of four squares that are connected with their closest neighbor at the vertices. In order to define the unit cell, two independent geometric parameters should be determined: the side length of each square, *l*, and the angle θ formed between two connected squares. The side lengths *X*_1_ and *X*_2_ of the unit cell can then be defined by the following equation

(17) $$\;X_{1}=X_{2}\;=\;2l\left[\cos\left(\frac{\theta }{2}\right)+\sin\left(\frac{\theta }{2}\right)\right]$$

Following the rotating square geometry, other shapes, such as rectangles, triangles, parallelograms, and rhomboidal systems have been reported as auxetic rotating topologies.^[^ ^67^, ^68^ ^]^ In the case of the rotating triangles, the unit cell is a parallelogram including two scalene triangles with sides *a*_1_, *b*_1_, *c*_1_ and *a*_2_, *b*_2_, *c*_2_, respectively. The interior angles of the two triangles are denoted by α_1_, β_1_, γ_1_ and α_2_, β_2_, γ_2_, where angle α_1_ is lying opposite to side *a*_1_, and so on.^[^ ^67^ ^]^ The angles formed at the hinges of the system are given by φ,  ω, and θ. The angles of the system are dependent variables, meaning that they can be described in terms of each other and the internal angles of the triangles through the following system of equations

(18) $$\left\{\begin{array}{c} \varphi \;=\;\theta -\alpha_{1}+\;\gamma_{2}=\;\omega +\beta_{1}-\alpha_{2}\\ \omega \;=\;\theta +\gamma_{1}-\;\beta_{2}=\;\varphi -\beta_{1}+\alpha_{2}\\ \theta \;=\;\omega -\gamma_{1}+\;\beta_{2}=\;\varphi +\alpha_{1}-\gamma_{2} \end{array}\right.$$

Using the above parameters, the shape and size of the unit cell can be defined as

(19) $$\;l_{1}=\sqrt{c_{1}^{2}+c_{2}^{2}+2c_{1}c_{2}\cos\left(\theta -\alpha_{1}-\beta_{2}\right)}\;\;$$

(20) $$\;l_{2}=\sqrt{b_{1}^{2}+b_{2}^{2}-2b_{1}b_{2}\cos\left(\theta +\gamma_{2}\right)}\;$$

(21) $$\alpha_{12}=\cos^{-1}\left(\frac{l_{1}^{2}+l_{2}^{2}-l_{3}^{2}}{2l_{1}l_{2}}\right)\;\;$$

The auxeticity of rotating rigids is attributed to the rotation mechanism at the connected vertices, when a uniaxial load is applied. Under compressive or tensile load, these structures are able to contract or expand, respectively. It is worth mentioning that rotating rigid systems have demonstrated an extensive range of Poisson's ratio values and isotropy, depending on the dimensions of their constituent shapes, the angles between them as well as the loading directions.^[^ ^67^ ^]^

Unlike the previously presented metamaterials, the constituent design elements of rotating rigids are not slender beams and ligaments but, as their name suggests, solid shapes. For tissue engineering, this translates to localized porosities of 0%, which could result in inhomogeneous tissue formation and scaffold/implant integration to the affected site. One approach to tackle this challenge would be to either decrease the size of the solid rigids or to introduce a porous infill in them. However, the infill geometry is expected to affect the final mechanical properties of the lattice and should, thus, be selected cautiously, in order to ensure the auxeticity of the design is not depleted.

#### 2.1.4 Bucklicrystals

**Figure 11:**
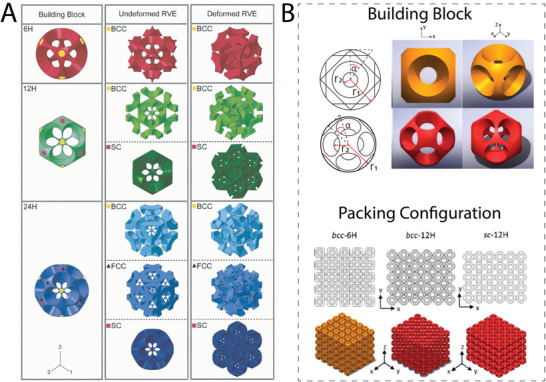
Bucklicrystal unit cells and packing configurations. A) Building blocks with 6, 12, and 24 holes. Undeformed representative volume elements (RVE) and buckle RVEs under uniaxial compression. Reproduced with permission.^[^ ^69^ ^]^ Copyright 2013, John Wiley and Sons. B) Top: building blocks with 6 holes and 12 holes. The design parameters *r*_1_, *r*_2_, and *α* are used to control the thickness and porosity. Bottom: packing configurations of the bcc‐6H, bcc‐12H, and sc‐12H unit cells. H: holes, bcc: body‐centered cubic, sc: simple cubic, fcc: face‐centered cubic. Adapted with permission.^[^ ^70^ ^]^ Copyright 2017, Elsevier.

Bucklicrystals are a class of 3D metamaterials that demonstrate a negative Poisson's ratio by contracting in the transverse direction upon compressive loading. They show isotropic reduction in volume under compression, as the elemental unit shell structure folds inward itself. This behavior is created by elastic, buckling‐based folding and was originally presented by Babaee et al.^[^ ^69^ ^]^ For the design of bucklicrystals, the starting unit cell is a patterned spherical shell. By controlling the arrangement of the holes on the shell, the subsequent volume change of the metamaterial can be tuned. Two bucklicrystal symmetries have been identified: octahedral symmetry, demonstrated by spherical shells with 6, 12, and 24 holes, and icosahedral symmetry, exhibited by spherical shells with 30 or 60 holes. The periodical arrangement of the spherical shells has been investigated in order to construct 3D body center cubic (bcc) and simple cubic (sc) structures (**Figure** **11**A). For the design of bucklicrystals, no universal geometric parameters have been provided, as their shape typically occurs upon studying the elastic instabilities of patterned spherical shells using computer‐aided simulations.^[^ ^69^ ^]^ Nevertheless, Yuan et al. reported three parameters, namely, *r*_1_, *r*_2_, and α, which were used to alter the porosity of the fabricated bucklicrystal blocks (Figure 11B).^[^ ^70^ ^]^

As the unit cells of bucklicrystals are based on 3D configurations, these metamaterials could be interesting for tissue engineering applications where space filling is of importance. For instance, they could be investigated as scaffolds for large bone defects or breast tissue regeneration. Additionally, the 3D interconnected porosity of the building blocks presented in Figure 11, indicates the potential for good tissue integration and vasculature support.

#### 2.1.5 Hybrid Auxetic Geometries

The exceptional mechanical properties of auxetic metamaterials have inspired researchers to propose new unit cell designs that could demonstrate a negative Poisson's ratio. The availability of a variety of additive manufacturing methods has largely contributed toward this research direction, by facilitating the fabrication of such experimental topologies. Here we include a selection of novel designs, aiming to inspire interested researchers to consider similar geometries in tissue engineering applications

**Figure 12:**
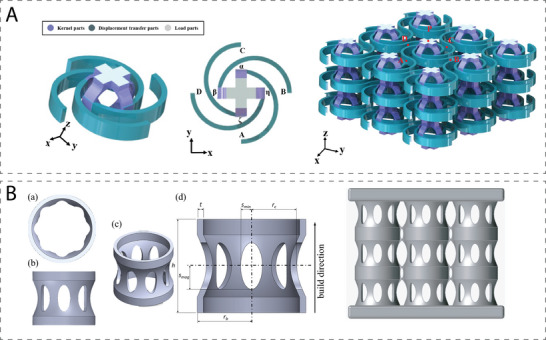
Examples of hybrid auxetic metamaterials. A) 3D unit cell and stacked configuration with auxetic behavior in both vertical and horizontal directions. Adapted with permission.^[^ ^71^ ^]^ Copyright 2023, Elsevier. B) Bucklicrystal‐like metamaterial based on a perforated hyperboloid enclosed in two rings. Adapted with permission.^[^ ^72^ ^]^ Copyright 2022, Elsevier.

Cui et al. presented a novel 3D unit cell for achieving a negative Poisson's ratio, in both the vertical and the horizontal directions.^[^ ^71^ ^]^ Three distinct regions of the unit cell were designed, namely, the kernel part, the displacement transfer part, and the loading platforms (**Figure** **12**A). The kernel part consists of eight orthogonally arranged curved rods at its core and the loading platforms are located on the top and bottom of it. The displacement transfer part is a structure with four arc‐like rods demonstrating a rotational symmetry around the core. A 2D auxetic unit cell was reported by Alomarah et al. who combined geometric features of re‐entrant and chiral honeycombs.^[^ ^73^ ^]^ This unit cell was subsequently used as a reference for the design of a more complex 3D construct. Uniaxial compression experiments revealed the auxetic behavior of 3D struts, which demonstrated Poisson's ratios lower than −1 for loading on all *x*, *y*, and *z* axes. In a different approach, Mizzi and Spaggiari used multi‐polygonal Euclidean tiling tessellations to define auxetic structures.^[^ ^74^ ^]^ Circular chiral nodes were designed at the vertices of the polygons and connected to each other by ligaments in the same arrangement. A host of different topologies could be obtained with this approach, introducing a “blueprint” for investigating new unit cell geometries.

The elliptic perforated plate has been used as the starting point for the development of a variety of novel unit cells.^[^ ^75^, ^76^ ^]^ Recently, a novel bucklicrystal‐like design was proposed by Galati et al., who reported the use of a thin, perforated hyperboloid enclosed in two rings (Figure 12B).^[^ ^72^ ^]^ The geometric parameters that are important for the design of this structure are the radii of the ring base and the thinner part of the paraboloid, *r_b_* and *r_c_*, respectively, the height of the whole unit cell, *h*, the thickness of the unit cell, *t*, and the semiminor and semimajor axes of the parabolic wholes, *s*_min_ and *s*_max_.

### 2.2 Ultraproperty Metamaterials

A desirable requirement for many structural biomaterials is that they exhibit both high strength and stiffness, for example, when the target tissue is bone. However, these two properties are mutually exclusive in most materials.^[^ ^77^ ^]^ Nevertheless, some natural materials, such as nacre and bone, show high mechanical performance, which has been attributed to their hierarchical and staggered microstructure.^[^ ^78^ ^]^ Following a similar design pattern, a class of ultraproperty metamaterials including ultrastiff, ‐strong, ‐tough, and ‐lightweight materials, emerged.^[^ ^79^, ^80^, ^81^, ^82^, ^83^ ^]^

**Figure 13:**
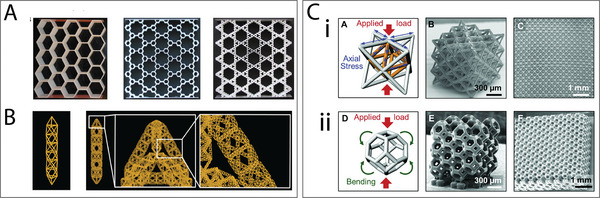
Ultraproperty metamaterials. A) Hierarchical honeycombs designed in a fractal‐like manner. The three first iterations of the replacement procedure are depicted. Adapted with permission.^[^ ^82^ ^]^ Copyright 2012, Elsevier. B) Second order fractal‐based scaffold based on a hollow beam building block. Reproduced with permission.^[^ ^83^ ^]^ Copyright 2017, EPLA. C) Architecture of stretch‐dominated and bend‐dominated unit cells and lattices.^[^ ^79^ ^]^ (i) Left: mechanical response to compressive loading of a stretch‐dominated octet‐truss unit cell. Middle: octet‐truss unit cells packed into a cubic microlattice. Right: SEM image of a stretch‐dominated lattice material composed of a network of octet‐truss unit cells. (ii) Left: mechanical response to compressive loading of a bend‐dominated tetrakaidecahedron unit cell. Middle: tetrakaidecahedron unit cell packed into a cubic bend‐dominated lattice Right: SEM image of a bend‐dominated lattice composed of a network of tetrakaidecahedron unit cells. Reproduced with permission.^[^ ^79^ ^]^ Copyrigh 2014, The American Association for the Advancement of Science.

Hierarchically arranged honeycombs were used by Ajdari et al. as a strategy to fabricate ultrastiff structures with significantly higher stiffness values, compared to that of their constituent materials.^[^ ^82^ ^]^ The design of the lattices was based on a replacement procedure that was repeated to different scales, showcasing the potential of fabricating fractal‐like honeycombs with higher orders of structural hierarchy (**Figure** **13**A). Mechanical tests on the hierarchical honeycombs revealed a 3.5‐fold increase in stiffness compared to the regular hexagonal honeycombs with the same mass, and illustrated that this topology could be used as a lightweight design for incompressible biomaterials.

An ultralight and strength‐resistant metamaterial was fabricated by Rayneau‐Kirkhope et al., based on a fractal design method, which resulted in constructs with increased order of structural hierarchy. In their approach, a hollow beam was considered as the building block of the geometry (named generation 0), which was used for the construction of a scaffold consisting of *n* octahedra and two tetrahedra. Using an iterative approach, the properties of the *n*th generation of the fractal structures were investigated. Although the study was mainly focused on larger constructs, a proof of concept for a generation‐2 scaffold with a total length of a few centimeters could be an intriguing starting point for tissue engineering applications (Figure 13B). Zheng et al. investigated octet‐truss and tetrakaidecahedron microlattices as a candidate topology for ultrastiff metamaterials (Figure 13C).^[^ ^79^ ^]^ The final metamaterials could maintain their ultrastiff properties across more than three orders of magnitude in relative mass density, regardless of the constituent material used. This extraordinary performance of the octet‐truss microlattices along with their complex porosity makes them excellent candidates for potential bone scaffolds, as they could be fabricated from a host of different materials while retaining their high stiffness.

An interesting method for the pyrolysis of additively manufactured face‐centered cubic lattices was recently reported by Surjadi et al.^[^ ^81^ ^]^ In this approach, a partial carbonization method was used to enhance the inherent high strength of the lattices. The pyrolyzed structures exhibited strength and energy absorption values that were 100 times higher than those measured for the noncarbonated controls. Moreover, the ultrastrong lattices could withstand loading up to a 50% strain without fracturing, which was almost double than the fracture strain of the controls, showcasing the extraordinary combination of high strength and ductility demonstrated by the partially carbonized microlattices.^[^ ^81^ ^]^

### 2.3 Origami‐ and Kirigami‐Inspired Metamaterials

The Japanese art of paper folding, origami and kirigami, has inspired the emergence of a new class of metamaterials that are based on folding deformations.^[^ ^84^ ^]^ Starting from a 2D, flat configuration, origami metamaterials can be folded or activated to self‐fold to a desired 3D shape.^[^ ^9^ ^]^ The final geometry of the 3D construct is governed by the design of the creases and the folding patterns. The kirigami approach shares the same design principles as origami, with the additional freedom of cuts/openings on the flat surface.^[^ ^85^ ^]^ The initial 2D configuration of both origami and kirigami design approaches allows the introduction of complex topographies on the folding “flaps” that could otherwise be challenging or even impossible to fabricate on a 3D construct.

**Figure 14:**
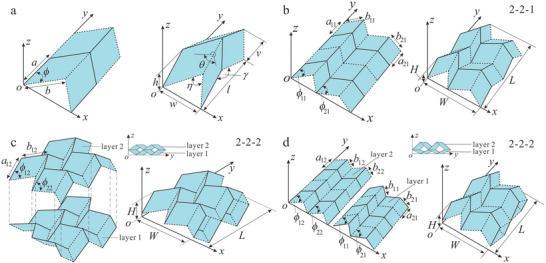
Miura‐ori unit cells and stacked configurations. Reproduced with permission.^[^ ^91^ ^]^ Copyright 2020, Elsevier.

Miura‐ori is the most widely researched type of origami structures. Although the Miura‐ori folding pattern is inspired by man‐made art, this morphology has been observed in insect wings, leaves and other laminae‐like organelles,^[^ ^86^ ^]^ as well as in embryonic intestine.^[^ ^87^ ^]^ Mechanical metamaterials designed based on the Miura‐ori, have been reported to exhibit negative Poisson's ratios and hold a great potential for use in tissue engineering.^[^ ^84^, ^88^, ^89^, ^90^ ^]^ The Miura unit cell can be defined as follows, using four independent parameters: the sides *a* and *b* of a parallelogram, the sector angle φ, and the folding angle θ (**Figure** **14**). Based on the above parameters, the partially folded state of the unit cell can be defined by the edge angles, η and γ, and the side lengths *w*,  *l*,  *h*,  and *v*, which are described by the following equations^[^ ^91^ ^]^

(22) $$\cos\gamma =\frac{{\left(\sin\varphi \cos\left(\frac{\theta }{2}\right)\right)}^{2}-\cos^{2}\varphi }{{\left(\sin\varphi \cos\left(\frac{\theta }{2}\right)\right)}^{2}+\cos^{2}\varphi }$$

(23) $$\cos\eta =\sin^{2}\varphi \;\cos\theta +\cos^{2}\varphi$$

(24) $$w=2b\sin\left(\frac{\eta }{2}\right)$$

(25) $$l=2a\sin\left(\frac{\gamma }{2}\right)$$

(26) $$h=\alpha \cos\left(\frac{\gamma }{2}\right)$$

(27) $$v=b\cos\left(\frac{\eta }{2}\right)$$

Based on the Miura unit cell, two metamaterial designs were presented by Schenk and Guest, a folded shell structure and a stacked configuration.^[^ ^88^ ^]^ The folded shell metamaterial, exhibits opposite Poisson's ratios for in‐plane and out‐of‐plane deformations. In other words, planar deformations result in a negative Poisson's ratio while bending yields a positive Poisson's ratio. Remarkably, the two ratios are equal and opposite. In the case of the stacked configuration, the fold pattern may differ between consecutive layers. In the proposed geometry, the unit cell design alternates between successive layers. To achieve the desired behavior, consecutive layers should share at least three geometric parameters.^[^ ^88^ ^]^ An interesting characteristic of the Miura unit cell is that when the maximum folding angle $\theta \;=\frac{\pi }{2}\;$ is reached, it self‐locks, and the folding of the entire sheet is halted. By programming the folding mechanism of the unit cell, bi‐ and multistable “metasheets” with tunable stiffness, shape, and size can be fabricated.^[^ ^89^, ^90^ ^]^

Kirigami‐based designs have been used for the fabrication of soft, and highly stretchable metamaterials.^[^ ^85^ ^]^ The transition from the stiff to the soft regime of the kirigami sheets had been attributed to the transition from 2D to 3D deformation. The softening metasheets presented by Isobe and Okumura can be defined by the length of the cuts *w*, the horizontal and vertical spacing *d* between the cuts and the number of cuts *N*. Based on those parameters, the sample height can be defined as 2*Nd*. Additionally, the constraint *w* < *d* is to be satisfied.^[^ ^85^ ^]^

The use of different kirigami designs has been proposed as a fabrication method for injectable stents. More specifically, unit cells of length *l*, cut angle γ, and hinge length δ, could deform into 3D needles that could be used for drug delivery into the gastrointestinal tract.^[^ ^92^ ^]^ Combining the aforementioned folding mechanisms with additive manufacturing techniques and other mechanical metamaterials that already possess superior properties may result in structures with enhanced and, perhaps, unprecedented features suitable for tissue engineering applications (e.g., flexible and deployable implants or active materials for drug‐delivering stents and scaffolds).

### 2.4 Next‐Generation Metamaterials: Artificial Intelligence‐Aided Metamaterial Design

Starting from the geometric parameters of the unit cell, a host of mechanical responses can be achieved solely by tuning the individual parameters or simultaneously altering multiple of their values. Therefore, depending on the tissue engineering application, there may be certain favorable combinations of individual parameters that yield the desired properties to the metamaterial. However, the number of conditions that needs to be tested can become significantly high. Thus, the challenge that arises is how to identify the optimal parameters of a given unit cell or, inversely, how to design a unit cell of optimal performance, depending on the application. The most common approach to tackle this issue is the use of finite element analysis (FEA) for predicting the mechanical response of the studied construct, under various loading scenarios, depending on the values of the geometric parameters of the unit cell. However, recently, artificial intelligence (AI) and machine learning (ML) approaches have emerged as promising tools that are expected to revolutionize the field of metamaterial design.^[^ ^93^, ^94^ ^]^ To the best of the authors’ knowledge, although AI and ML have been used for the design of tissue engineering scaffolds,^[^ ^95^ ^]^ they have not yet been applied to metamaterial design targeting specific tissues. Nevertheless, they are expected to greatly accelerate the broader use of complex, metamaterial‐based geometries by making their design more accessible to researchers of various backgrounds.

**Figure 15:**
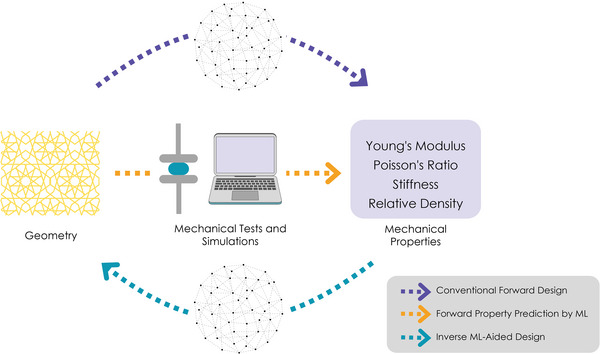
Overview of forward and inverse design approaches. ML: machine learning. Created with Rhino7 and BioRender.com.

AI and ML could significantly upgrade the metamaterial design process, in combination with FEA, by predicting potential errors, thus simultaneously reducing the required computational load.^[^ ^96^, ^97^ ^]^ Based on the computational results, machine learning could also be used for inverse design of new unit cells that would demonstrate a user‐defined mechanical response when loaded (**Figure** **15**).^[^ ^94^ ^]^ More specifically, models that accept loading conditions and desired mechanical responses as input, and generate a unit cell geometry exhibiting this exact behavior, have recently been reported in the literature.^[^ ^98^ ^]^

By modifying the auxetic angle of re‐entrant honeycomb cells, Wilt et al. could change the auxetic response of metamaterial lattices and use the results as metrics for ML input selection.^[^ ^96^ ^]^ Using the prediction results from the ML model, a design with a low predicted mean average error could be selected. Experimental results demonstrated that the behavior of the selected geometry was similar to the simulation results.^[^ ^96^ ^]^ Meyer et al. presented a deep‐learning method of constructing metamaterial lattices based on a graph representation approach. Starting from triply periodic minimal surface topologies the model was used to create more than 43 000 distinct lattices and predict their thermal and elastic properties using FEA. Furthermore, the model could be used for the solution of the inverse problem, i.e., to discover lattice topologies capable of providing targeted macroscopic properties.^[^ ^98^ ^]^ Given the widespread use of additive manufacturing in metamaterials research, new ML models for inverse metamaterial design could also be expanded to take the fabrication method into account. For instance, a novel ML model was presented by Ha et al. that was capable of designing unit cells, while simultaneously predicting their compressive response and account for potential fabrication‐related flaws.^[^ ^99^ ^]^

Algorithmic approaches have been proposed for the inverse design of origami and kirigami geometries to precisely fit a 2D or 3D boundary.^[^ ^100^, ^101^, ^102^ ^]^ Choi et al. presented a solution to the inverse problem of determining the number, size, and orientation of cuts on a kirigami plane to enable its deployment in any 2D or 3D space. Experimental validation of the inverse design with physical kirigami models revealed the potential of implementing this novel approach for the fabrication of shape‐morphing mechanical metamaterials.^[^ ^102^ ^]^ A versatile, computational approach for designing origami mechanisms of arbitrary size and complexity was developed based on the principle of three units for rigid foldability. A variety of complex geometries was designed and fabricated using both paper as well as 3D printed parts.^[^ ^101^ ^]^

## 3 Mechanical Metamaterials in Tissue Engineering

The use of metamaterials for restoring or improving the properties of native tissues is a newly emerged field of research. Given the surge of published studies on novel metamaterials and inverse design, applications of metamaterial geometries in tissue engineering are following a more conservative trend. Nevertheless, mechanical metamaterials could hold a great potential for tissue regeneration. As discussed above, specific native tissues exhibit negative Poisson's ratios upon loading.^[^ ^33^, ^34^, ^35^, ^36^ ^]^ Hence, auxetic designs could be considered for fabricating scaffolds that closely match the mechanical response of the target tissue in vivo. Moreover, mechanical metamaterials exhibit interesting deformation behaviors under complex loading regimes. Therefore, rationally designed materials could be good candidates for the fabrication of patches or thin lattices destined for tissues that undergo large strains, such as the skin,^[^ ^103^, ^104^, ^105^, ^106^ ^]^ and the heart.^[^ ^107^, ^108^, ^109^ ^]^ Furthermore, auxeticity could significantly facilitate the introduction and integration of tubular structures, such as stents and vascular grafts to the affected site. On the other hand, ultraproperty metamaterials could be considered for the regeneration of tissues with complex mechanical responses that cannot be found in conventional materials, i.e., the tendons.^[^ ^23^ ^]^ In the following sections we summarize the current mechanical metamaterial applications in tissue engineering, classified per tissue type. An overview of all the reviewed studies can be found in **Table** **2**.

**Table 2:** Overview of metamaterial applications in tissue engineering. Dots (●) and dashes (−) represent the presence or absence of geometric parameter analysis and simulations from the reviewed studies, respectively. TO stands for Topology Optimization.

Target tissue		Metamaterial type	Unit cell geometry	Material	Fabrication	Poisson's ratio	Parameter analysis	Simulations	Biological experiments	Study
Bone		Auxetic	Double arrowhead	CoCrMo	Laser powder bed fusion	−0.037	●	●	–	[111]
			Re‐entrant honeycomb Arrowhead Modified re‐entrant Double arrowhead Bell‐shaped	CoCrMo	Laser powder bed fusion	−0.24 to −0.1	–	–	–	[110]
			Re‐entrant honeycomb with added patches	Ti‐6Al‐4 V ELI	SLM	−0.33 to −0.108	–	●	–	[113]
			Re‐entrant honeycomb‐like	PLGA	Solvent casting and permanent volumetric compression	−0.07 to −0.01	–	–	In vitro human osteoblast‐like cells	[121]
			Connected stars	PCL	MEW	Graph of transverse versus longitudinal strain	●	–	In vitro HUVECs and BMSCs	[114]
		Nonauxetic	Diamond Body‐centered cubic Rhombic dodecahedron	Cp‐Ti	SLM	From 0.5 to ≈1.5 depending on geometry and relative density	●	–	–	[112]
Cardiovascular	Cardiac patches	Auxetic	Orthogonal missing rib Re‐entrant honeycomb Rotated re‐entrant honeycomb	PCL	DIW	Graph of transverse versus longitudinal strain	●	●	In vitro hIPSC‐CMs	[107]
			Re‐entrant honeycomb	Polyaniline on chitosan	Excimer laser microablation	Anisotropic ratio of effective stiffness used instead	●	–	Ex vivo rat hearts In vivo rat MI model	[108]
			Square grid (missing rib)	PPy‐coated PCL	MEW	−1.0 to 0.0	–	–	In vitro hMSCs Ex vivo rat skeletal muscle	[109]
	Vascular stent	Auxetic	Rotating squares	PCL	MEs, pulse laser ablation, and welding	−1.02	–	–	–	[131]
				–	–	−1 (theoretical)	●	–	–	[130]
			Anti‐trichiral	TPU and SS	Laser cutting	−2.2 (most negative value measured)	●	●	–	[134]
			Anti‐tetrachiral	Photocurable polymer coated with Au nanofilm	PµSL	Not included	–	–	In vitro hMSCs and human foreskin fibroblasts	[135]
				SS	Laser cutting	−5.8601 to −0.8295	●	●	–	[136]
			Tetrachiral	Vero and Tango	Polymer jetting	−0.82 (theoretical)	TO	●	–	[133]
			Arrowhead	PLA	DIW	Not included	●	–	–	[140]
			Re‐entrant honeycomb	PLA	Twin‐screw‐based printing	Not included	–	●	Ex vivo sheep intestine	[139]
			Hexachiral	SS	Laser cutting	−0.69 (for the optimal geometry)	–	●	–	[132]
			Peanut‐shaped hole	Vero white plus	Jet printing	−0.805	●	●	–	[142]
			Ring‐link	Ninjaflex	FDM	Binary state: −0.674 to 0.679 Continuous state: −0.536 to 0.532	●	●	–	[141]
			Hybrid: anti‐trichiral/re‐entrant honeycomb	SS	SLA	−1.3121 to −0.8524	●	●	–	[143]
			Hybrid: tetrachiral with re‐entrant node Hybrid: tetrachiral with star node	Nitinol (for simulations)	–	(−0.2,−0.15)	–	●	–	[144]
			Hybrid: tetrachiral/anti‐tetrachiral	Nylon	SLS	−2.5 to −0.3	●	●	–	[145]
			Novel	SS	–	−0.315 to −0.309	–	●	–	[146]
		Origami	Miura‐ori	TiNi	Etching	Not included	–	–	–	[147]
		Ultraproperty	Lightweight/ultratough	PEGDA	DLP followed by pyrolysis	Not included	–	–	In vitro invasive breast cancer cells	[81]
	Vascular graft	Auxetic	Square grid (missing rib)	PEGDA	Projection‐based printing	−0.6 to −0.1	–	●	In vitro human turbinate MSCs	[137]
			Re‐entrant honeycomb	PCL	Electrospinning and DIW	Single layer: −0.551 to −0.401 Multilayer: −0.750 to −0.533	–	–	In vitro HUVECs and human VSMSCs	[138]
	Cardiac pump‐on‐a‐chip	Auxetic	Inverted hexagon	IP‐S	TPDLW	Not included	–	●	In vitro hiPSCs‐CMs	[127]
Skin	Wound dressing	Auxetic	Elliptical and rectangular holes	HEMA	DLP	Not included	–	●	–	[105]
			Zigzag lattice	VeroBlue	Jet printing	−1 to 1	–	●	–	[106]
		Ultraproperty	Sinusoidal	Liquid crystal elastomer	Laser cutting	Not included	–	●	In vitro rat fibroblasts In vivo rat wound model	[103]
	Hypertrophic scar treatment	Auxetic	Re‐entrant honeycomb Double arrowhead	TPU	FDM	−1.3 to −0.75	●	●	–	[104]
Neural		Auxetic	Re‐entrant honeycomb‐like	PU	Triaxial compression	−0.45	–	–	In vitro ES‐D3 and human iPSK3	[179]
			Rotating squares	GelMA	Molding	Not included	–	–	In vitro human Schwann cells	[178]
Oesophagus	Oesophageal stent	Auxetic	Rotating squares	PU	Laser cutting	−0.76 to −0.72	–	–	–	[164]
					Vacuum casting	−0.96 to −0.87	–	●	–	[166]
				Polypropylene	Laser cutting	−0.91 to −0.89	–	–	–	[165]
Tendon		Ultraproperty	Strengthened rectangular‐cuboid inclusions	Resin	Microfabrication STL	1 to 8	●	●	–	[23]
Myotendinous junction		Auxetic	Re‐entrant honeycomb	PU	Dynamic Optical Projection Stereolithography	Not included	–	●	In vitro fibroblasts and myoblasts	[24]
Trachea		Auxetic	Hybrid: tetrachiral/anti‐tetrachiral	Silicone	Molding and salt leaching	−0.3 to 0.1	●	●	In vitro human bronchial epithelial cells Ex vivo pork tracheas	[169]
Intervertebral disc		Bucklicrystal	Hollow shell cuboctahedron	TPU	SLS	−0.52 to −0.33	–	●	In vitro rabbit chondrocytes and nucleus pulposus cells In vivo rabbit disc replacement model	[186]
		Auxetic	Re‐entrant honeycomb	Polyethylene	Milling	−1 to −0.6	–	●	–	[185]
Vertebrae		Origami, kirigami	Novel	PLA, aluminum, titanium	FDM, laser cutting, laser micromachining	Not given	–	–	–	[22]
Cellular level		Auxetic	Re‐entrant honeycomb	PEGDA	TPP	−1.5 to 0	–	–	In vitro 10T1/2	[187]
				SZ080	Multiphoton lithography	Not included	–	●	In vitro mouse fibroblasts	[189]
			Hybrid: tetrachiral/re‐entrant honeycomb	Silicon	Deep reactive ion etching	Not included	–	●	In vitro hMSCs	[188]
			Re‐entrant honeycomb Hybrid: re‐entrant honeycomb with conventional honeycomb	Methacrylate‐based photoresist (IP‐Q)	2PP	−0.74 to 0.74	–	●	In vitro mouse preosteoblasts	[190]
			Re‐entrant honeycomb	Cellulose nanofibers/PEGDA aerogels	SLA	Not included	–	–	In vitro mouse BM‐MSCs	[191]

### 3.1 Bone

Perhaps unsurprisingly, bone is one of the most researched target tissues for mechanical metamaterial applications.^[^ ^102^, ^110^, ^111^, ^112^, ^113^, ^114^ ^]^ Bone is a tissue with remarkable mechanical properties that vary from auxetic to ultrastiff, depending on the anatomical sight and bone type considered. The macroscopic mechanical response of bone depends on the level of porosity, ranging from cortical bone (with a porosity between 5% and 30% and a compressive strength of 100–230 MPa) in the cortex to cancellous bone in medullary cavities (with a porosity between 30% and 90% and a compressive strength of 2–12 MPa). Moreover, bone possesses seven levels of structural hierarchy, which are responsible for its extraordinary toughness, especially in the case of cortical bone.^[^ ^115^ ^]^ When loaded, cortical bone exhibits two toughening mechanisms: intrinsic, at the scale of tropocollagen molecules and mineralized collagen fibrils due to molecular uncoiling and intermolecular sliding, and extrinsic, at a scale of tens to hundreds micrometers, which arises from crack deflection and bridging.^[^ ^77^ ^]^ Additionally, bone is remodeled in response to its local loading environment, with bone that is not being loaded getting resorbed by osteoclasts.^[^ ^116^ ^]^ Thus, the ideal implants for bone regeneration should possess a high porosity, have a hierarchical structure and respond to local loading in a way similar to native tissue, in order to avoid the resorption of newly formed bone. Therefore, cellular metamaterials could be a superior candidate for bone implant design.^[^ ^117^ ^]^

#### 3.1.1 Auxetic Scaffolds

**Figure 16:**
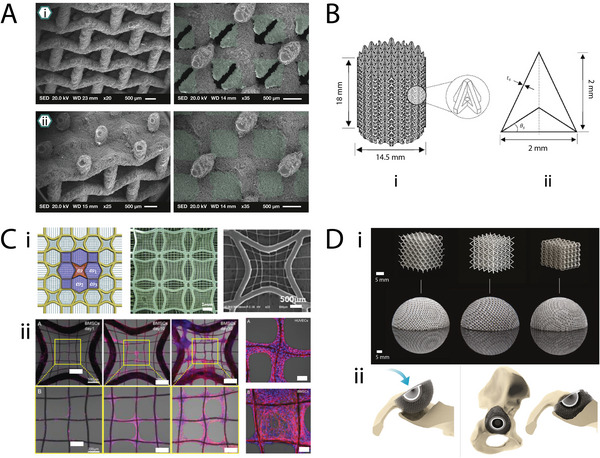
Mechanical metamaterial applications in bone tissue engineering. A) Scaffolds based on a 3D re‐entrant honeycomb configuration, fabricated from titanium alloy powder (SEM images). The auxetic lattices were combined with minimal surface patches (i) or fully covered on the vertical side (ii) (green pseudocolor). Scale bars: 500 µm. Reproduced under the terms of the CC‐BY license.^[^ ^113^ ^]^ B) (i) An auxetic bone scaffold design based on a double arrowhead unit cell, with dimensions suitable for the reconstruction of a critical‐size tibia defect. (ii) The unit cell dimensions on one of the symmetry planes. Reproduced under the terms of the CC‐BY license.^[^ ^110^ ^]^ C) (i) Left: design of melt‐electrowritten interconnected star‐based scaffolds bridges with electrospun fibers. The different re‐entrant angles present in the scaffolds are noted with *ω*, *ω*1, *ω*2, *ω*3. Middle: optical microscope image of the full scaffold. Scale bar: 1 mm. Right: SEM image of a star‐shaped region of the scaffold. Scale bar: 500 µm. (ii) Left: confocal laser‐scanning images of BMSCs cultured on the scaffolds for 1, 10, and 30 days. Scale bars: 500 µm (top row), 200 µm (bottom row). Right: confocal laser‐scanning images of HUVECs and BMSCs on Day 30. Scale bars: 100 µm. Reproduced with permission.^[^ ^114^ ^]^ Copyright 2020, Elsevier Ltd. D) (i) Nonauxetic, space filling, femoral metaimplants based on the diamond (left), body‐centered cubic (middle), and rhombic dodecahedron unit cell (right). Scale bars: 5 mm. (ii) Illustration of the space‐filling capacity of the implants to restore the physiological loading conditions of the femur. Reproduced under the terms of the CC‐BY license.^[^ ^112^ ^]^

In bone tissue engineering, the surface area of scaffolds or implants is crucial to cell adhesion and proliferation as well as the mechanical properties of the constructs.^[^ ^118^ ^]^ Nevertheless, increasing the surface area of a scaffold typically has a direct effect on the mechanical properties of the specimen. Addressing this challenge, auxetic 3D lattices enhanced with minimal surface patches were proposed as a strategy for decoupling the lattice surface area from its mechanical properties.^[^ ^113^ ^]^ Scaffolds based on the conventional and re‐entrant honeycomb unit cells, respectively, were fabricated from a titanium alloy powder using selective laser melting. Combining those lattices with minimal surface patches resulted in an increase of the total surface of the specimens, which is favorable for osseointegration,^[^ ^119^ ^]^ without, however, significantly affecting their mechanical properties (**Figure** **16**A). This strategy may be used for tuning the permeability and degradation rate of bone implants and scaffolds, but could also be considered as a control mechanism for drug release kinetics.^[^ ^120^ ^]^

Based on the structural similarities between bone and cellular metamaterials, the latter have been studied for their potential use in critical size bone defects. Wanniarachchi et al. investigated five unit cell geometries to fabricate 3D lattices from cobalt–chromium–molybdenum with near zero or negative Poisson's ratio, for bone tissue engineering.^[^ ^110^ ^]^ The resulting scaffolds exhibited Poisson's ratios ranging from −0.24 to −0.1 and elastic moduli in the 4.85–8.49 GPa range. Five criteria (Poisson's ratio, porosity, yield strength, elastic modulus, and ease of design) were selected and used to fabricate a decision‐making matrix, which could provide the optimal geometry based on the site of interest. The output of the model identified the 3D re‐entrant honeycomb as the predominant candidate for bone scaffolds with negative Poisson's ratio, while the 3D arrowhead unit cell could be selected for bone applications requiring a near‐zero Poisson's ratio scaffold (Figure 16B). The double‐arrowhead auxetic geometry was further explored in a later study by the same group, in order to create “stiffness‐matching” scaffolds with tailored properties for load‐bearing tissue engineering purposes.^[^ ^111^ ^]^ A surrogate framework was implemented for the estimation of the trends of Poisson's ratio, porosity, strength, and elastic modulus as functions of geometric parameters of the unit cell, including the strut thickness and the auxetic angle. Based on the surrogate model, design and fabrication of personalized scaffolds that closely match the properties of a patient's native tissue could be greatly facilitated. In both works, a significant focus was placed on predicting the optimal metamaterial geometry depending on the requirements of the application or the patient, showcasing the versatility of mechanical responses that can be achieved solely by tuning the unit cell configuration.

Although yet limited, in vitro studies of metamaterials in bone tissue engineering have explored the potential of combining extraordinary geometries with cells. Choi et al. used the triaxial compression approach first described by Lakes,^[^ ^27^ ^]^ to fabricate scaffolds from poly(lactic‐*co*‐glycolic acid) (PLGA) foams with negative Poisson's ratio. Human osteoblast‐like cells (MG‐63) were seeded in the scaffolds and the auxetic foams were cultured for 5 days under dynamic mechanical compression. The proliferation rate of the osteoblasts was higher for the first 3 days of culture in the dynamically loaded auxetic foams compared to the static controls. However, no significant differences were observed on day 5.^[^ ^121^ ^]^ Auxetic polycarpolactone (PCL) scaffolds based on the UC#01, UC#02, and UC#03 star grids^[^ ^46^ ^]^ were fabricated by Jin et al. via melt electrowriting.^[^ ^114^ ^]^ The pores of the auxetic lattices were bridged by thinner electrospun fibers, in order to promote cell adhesion and growth (Figure 16C(i)). The re‐entrant angles of the stars were varied from 110° to 140° and heterogeneous grids were designed by combining different unit cells. Uniaxial tensile tests indicated that the modulus increased from 40 to 100 MPa with an angle increment of 10°, illustrating the importance of controlling the independent unit cell parameters. The multiscale scaffolds were further assessed for their biocompatibility, by seeding human umbilical cord endothelial cells (HUVECs) and bone marrow‐derived stem cells (BMSCs) (Figure 16C(ii)). Although both cell types populated the scaffolds and remained viable for 30 days, no mechanical stimulation was provided. Hence, the active effect of the auxetic geometry was not investigated further in this study.

#### 3.1.2 Nonauxetic Metaimplants

Apart from the matching of the mechanical properties, the space‐filling capacity of bone scaffolds is essential for effective support and tissue regeneration. Kolken et al. investigated the space‐filling behavior of nonauxetic, functionally graded metaimplants for the treatment of critical size acetabular bone defects.^[^ ^112^ ^]^ Three nonauxetic unit cell geometries, namely, the diamond, the body‐centered cubic, and the rhombic dodecahedron, were chosen to be incorporated in the deformable implants, due to their high Poisson's ratios and low elastic moduli (Figure 16D). A relative density gradient was used in all three cases starting from 10% on the inner part to 2–4% on the outer part, in order to enhance the deformability of the implants. The functionally graded, diamond‐based implants showed the most favorable deformation and space‐filling properties, which were found to surpass common filling materials in ductility and strength, and could potentially be used to create long‐lasting, deformable bone implants in the future. It is worth mentioning that the incorporation of a functional gradient in the space‐filling metaimplants could also act as a cue for osteogenesis.^[^ ^122^ ^]^ However, the biological effects of the specimens were not investigated in this study.^[^ ^112^ ^]^

Summarizing the reviewed studies, it becomes apparent that metamaterials meet key requirements for bone scaffold design, as they have been shown to i) provide mechanical responses similar to native bone, ii) expand upon loading to effectively fill the defect or implantation site, and iii) provide sufficient adhesion surface for tissue integration. Moreover, decision models for matching the properties of a specific patient's tissue have been developed and could significantly aid toward the clinical translation of metamaterials for bone regeneration. Nevertheless, more studies are needed to better understand the effect of active mechanical metamaterial stimulation on stem cell differentiation and bone tissue formation.

### 3.2 Cardiovascular Tissue

#### 3.2.1 Myocardium

Heart is a mechanically active organ possessing the extraordinary ability of rhythmic contraction, which can be adjusted to the circulatory demand of the body on a beat‐by‐beat manner.^[^ ^123^ ^]^ The stiffness of the myocardium is instrumental for the mechanical and electrical function of the heart. For instance, increased myocardium stiffness restricts the speed and extent of diastolic relaxation, which, in turn, affects the chamber filling and limits the contraction velocity of cardiomyocytes. Moreover, stiffness is one of the major factors that will determine the outcome of a healing myocardial infarction (MI), from catastrophic rapture, necrotic or fibrotic tissue formation to remodeling.^[^ ^124^ ^]^ Since myocardium is an anisotropic tissue and its mechanical properties are directionally dependent, its Poisson's ratio can exceed 0.5.^[^ ^123^ ^]^ Negative Poisson's ratios have not yet been reported for cardiac tissue. However, auxeticity holds promising potential for cardiac tissue engineering and has already been investigated for the generation of cardiac patches.^[^ ^107^, ^108^, ^109^ ^]^

##### Auxetic Patches and Ventricles

**Figure 17:**
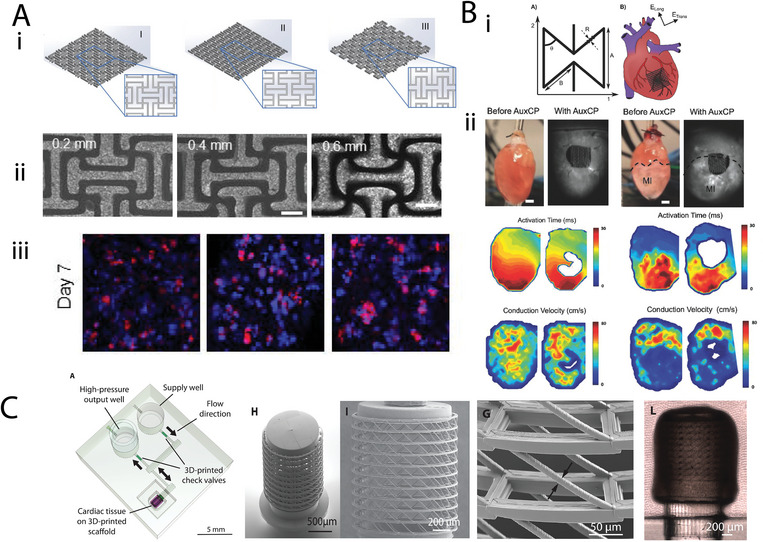
Mechanical metamaterials applications for myocardium and ventricle tissue engineering. A. (i) Three auxetic geometries investigated for the fabrication of cardiac patches. Left: missing rib, middle: re‐entrant honeycomb, right: rotated re‐entrant honeycomb. (ii) Bright‐field images of iPSC‐cardiomyocytes distribution in auxetic patches of different strut thicknesses at day 7. Scale bar = 500 µm. (iii) The auxetic patches laden with iPSC‐CMs stained against cardiac troponin T (red) and nucleus (blue) at day 7. Scale bar = 100 µm. Reproduced under the terms of the CC‐BY license.^[^ ^107^ ^]^ B) (i) Schematic illustration of the re‐entrant honeycomb scaffold as a cardiac patch. (ii) Activation time maps and CV maps before and after auxetic cardiac patch application on healthy hearts and hearts two weeks after MI. Reproduced under the terms of the CC‐BY license.^[^ ^108^ ^]^ C) Left: schematic of the complete ventricle system with the auxetic chamber. Middle: SEM images of a chamber scaffold based on the auxetic inverted hexagon unit cell and a helical scaffold design (bottom) to avoid chamber collapse due to tissue contractions. Right: tissue growth and shape integrity of the auxetic chamber. Reproduced with permission.^[^ ^127^ ^]^

Patches fabricated from metamaterial lattices can withstand large, out‐of‐plane, cyclic deformation without fracturing, while exhibiting high shock and energy absorption, hence closely matching the function of native myocardium. Cardiac metamaterial patches could be considered for the treatment of MIs by providing structural and functional support to the infarct tissue, while mechanically coupling it to the surrounding healthy myocardium, during tissue remodeling. This hypothesis was explored by Brazhkina et al. who proposed the use of auxetic patches for improving the cardiac function post‐MI.^[^ ^107^ ^]^ The 3D printed lattices, which were based on an orthogonal, missing‐rib unit cell geometry, exhibited lower elastic moduli compared to their hexagonal re‐entrant honeycomb counterparts, closely imitating physiological values of healthy cardiac tissue under similar loading conditions (0.2–0.5 MPa) (**Figure** **17**A(i)). The patch thickness was found to affect the overall stiffness of the construct, without influencing its auxetic behavior. This finding indicates a possible strategy for adjusting the properties of the patches to match the exact stiffness of the patient myocardium, while preserving their ability to perform in the auxetic regime. In vitro experiments indicated that human induced pluripotent stem cell‐derived cardiomyocytes could successfully attach and proliferate in the patches without losing their functionality for a 14‐day period (Figure 17A(ii,iii)).^[^ ^107^ ^]^

Besides the stiffness alterations accompanying an MI, the propagation of electrical impulses through the affected tissue is severely hampered, resulting in an arrhythmic inhomogeneous electrical distribution.^[^ ^125^ ^]^ Therefore, restoration of the electrical activity of the heart is vital in the restoration of normal cardiac function.^[^ ^126^ ^]^ Hence, combining conductive materials with auxetic geometries could yield cardiac patches with superior properties for MI treatment. Auxetic cardiac patches based on the lozenge (or missing rib) unit cell, demonstrating electroconductive properties close to those of healthy human myocardium, were presented in a recent study.^[^ ^109^ ^]^ Tensile tests on the patches revealed that they could maintain a negative Poisson's ratio for up to 40% and 60% strain in the transverse and longitudinal direction, respectively. The auxetic patches supported electrical conduction as a bridging construct between two hind limb rat muscles during ex vivo experiments and showed good cytocompatibility 48 hours after being seeded with neonatal cardiac fibroblasts. In another study, a conductive, auxetic patch was fabricated using excimer laser microablation on chitosan films and used for *ex vivo* and in vivo experiments for the treatment of infracted rat heart tissue.^[^ ^108^ ^]^ The cardiac patch was based on the re‐entrant honeycomb unit cell and its mechanical properties could be tuned to match those reported for native heart tissue, solely by altering its independent geometric parameters (Figure 17B(i)). *Ex vivo* experiments demonstrated that the conductive, auxetic patches have no detrimental effect on the electrophysiology of both healthy and MI rat hearts. However a reduced tissue contractility was reported (Figure 17B(ii)). Furthermore, the patches were observed to successfully integrate with native heart tissue in a rat MI model for over two weeks in vivo.

In a different approach, a miniaturized metamaterial scaffold capable of supporting the anisotropic contraction of an in vitro cardiac ventricle was presented by Michas et al.^[^ ^127^ ^]^ The scaffold was incorporated in a microfluidic device, which successfully recapitulated the unidirectional volumetric output of the human ventricle. The chamber was fabricated using two photon direct laser writing and was based on an auxetic inverted hexagon unit cell. The artificial ventricle could support dense tissue formation around it, 4 days after hiPSC‐CMs seeding, and was observed to contract in a cyclic manner, due to the beating of the cardiac tissue (Figure 17C).

In summary, auxetic geometries have been investigated for cardiac patch design demonstrating i) mechanical responses matching those of native myocardium, ii) sufficient conductivity, and iii) support of cardiomyocyte viability both in vitro and in vivo. So far, only two types of metamaterial geometries have been presented in the literature, the missing‐rib and re‐entrant honeycomb grids. More geometries could be explored both for their mechanical performance under contractile loading as well as for their electrical conduction. It should, however, be noted that a crucial prerequisite for proper function of cardiomyocytes is their organization, which can be greatly affected by topological cues.^[^ ^128^ ^]^ Therefore, the complex unit cell geometries could also be further evaluated on their effect on cardiomyocyte alignment.

#### 3.2.2 Vascular Stents

Intravascular stents are tubular structures that are introduced into stenotic arteries in order to restore blood flow perfusion.^[^ ^129^ ^]^ Typically, stents are delivered to the stenotic site mounted on a balloon catheter. When the balloon is inflated, the stent expands against the inner wall of the artery. Upon the balloon deflation, the stent should remain in place without recoiling, keeping the artery open. Therefore, the mechanical performance and, by extent, the geometry of the stent are instrumental to its performance. In terms of host tissue mechanics, arterial endothelium experiences a shear loading due to blood flow as well as a cyclic radial strain due to pulsatile flow. Interestingly, the thickness of the sub‐endothelial fiber layers in bovine carotid arteries have been observed to increase with radial strain, thus demonstrating an auxetic behavior.^[^ ^37^ ^]^

The negative Poisson's ratio exhibited by auxetic metamaterials has attracted a considerable research interest in the field of intravascular stent engineering, as it can facilitate both the introduction and deployment of the devices, while closely matching the mechanical properties of the surrounding vascular tissue. Vascular stents have been designed based on rotating rigids,^[^ ^130^, ^131^ ^]^ chiral,^[^ ^132^, ^133^ ^]^ and antichiral unit cells,^[^ ^134^, ^135^, ^136^ ^]^ missing rib/lozenge lattices,^[^ ^137^ ^]^ re‐entrant honeycombs,^[^ ^138^, ^139^ ^]^ arrowheads,^[^ ^140^ ^]^ ring‐link configurations,^[^ ^141^ ^]^ peanut‐shaped holes,^[^ ^142^ ^]^ hybrid geometries,^[^ ^143^, ^144^, ^145^ ^]^ as well as novel designs based on topology optimization methods.^[^ ^146^ ^]^ Apart from auxetic metamaterials, origamis^[^ ^147^ ^]^ and ultramaterials^[^ ^81^ ^]^ have also been investigated for their potential in the fabrication of vascular stents.

##### Auxetic Stents

**Figure 18:**
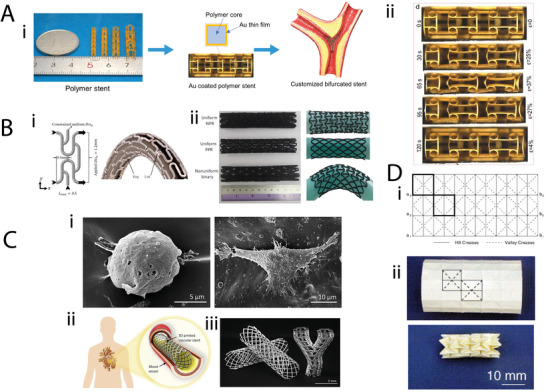
Mechanical metamaterials applications for vascular stents fabrication. A) (i) Manufacturing process of the thin‐walled antichiral stents. (ii) Deformation of the polymer stents through Au thin film coating for increasing strain values. Reproduced with permission.^[^ ^135^ ^]^ B) (i) CAD of ring‐and‐link unit cells for the fabrication of stents for curved arteries. (ii) Stents with uniform negative Poisson's ratio, uniform positive Poisson's ratio, and nonuniform binary Poisson's ratio before (left) and after expansion by a balloon catheter (right). Reproduced with permission.^[^ ^141^ ^]^ C) (i) SEM images of cell adhesion on the as‐fabricated and partially carbonized microlattices at different magnifications. (ii) Illustration of the potential application and (iii) images of functionally coated core–shell coronary stents with partially carbonized core. Scale bar: 5 mm. Reproduced with permission.^[^ ^81^ ^]^ D) (i) Illustration of the design of the origami stent. Continuous lines indicate expected hills while dotted lines show the valley creases. (ii) Images of the folded (left) and deployed origami stent (right). Reproduced with permission.^[^ ^147^ ^]^

In one of the earliest studies on auxetic stents, PCL patches based on the rotating square pattern were fabricated by combining melt electrospinning with micromachining by pulse laser ablation.^[^ ^131^ ^]^ The flat patches exhibited a Poisson's ratio of ≈−1.02 and were subsequently processed into tubular stents by welding. Triangular anti‐trichiral stents were introduced as a superior design in terms of uniform shrinkage and high energy absorption under compressive loading.^[^ ^134^ ^]^ Simulations and mechanical tests revealed that the novel anti‐trichiral design could shrink uniformly, with a maximum Poisson's ratio value of −2.2 on the *xz* plane and −2.19 on the *xy* plane. Compared to stents designed with the typical anti‐trichiral unit cell,^[^ ^59^ ^]^ a threefold increase in both stiffness and energy absorption capability were observed for the triangular anti‐trichiral stents. Moreover, the anti‐trichiral unit cell has been combined with the re‐entrant honeycomb to produce hybrid lattices that were used for the fabrication of vascular stents.^[^ ^143^ ^]^ The mechanical properties of the stents were found to be dependent on the unit cell geometry and ligament thickness. Simulation and experimental results indicated that proper selection of the unit cell geometry resulted in minimal radial recoil and foreshortening. An anti‐tetrachiral stent was recently developed to address the issue of low radial strength in thin‐walled 3D printed stents (**Figure** **18**A(i)).^[^ ^135^ ^]^ The thin‐walled stents were fabricated using high‐resolution projection micro‐stereolithography (PµSL) from a biodegradable, photocurable polymer and subsequently subjected to gold nanofilm sputtering (≈100 nm).^[^ ^135^ ^]^ Stent coating resulted in higher elastic moduli and yield strength values under radial compression, regardless of the initial thickness of the specimens (Figure 18A(ii)). Moreover, thicker stents (≈250 µm) were observed to fully recover their initial shape upon 35% radial compressive strain, while the gold nanocoating exhibited no obvious cracks or delamination. Cytocompatibility experiments revealed high viability rates of human foreskin fibroblasts and human mesenchymal stem cells (hMSCs) cultured for 5 days on the stents. A similar stent design has also been suggested by Wu et al. who analyzed the in‐plane mechanical performance of anti‐tetrachiral stents with circular and elliptical nodes as well as a fractal‐like, hierarchical anti‐tetrachiral stent architecture.^[^ ^136^ ^]^ It was reported that the mechanical properties of the stent could be tailored by adjusting the independent design parameters of the unit cell and the level of hierarchy. Simulations of the stent‐plaque‐artery system revealed that the studied geometries could exhibit remarkable radial expansion while maintaining their axial stability.

An auxetic coronary stent design for recoil optimization after the catheter balloon deflation was proposed.^[^ ^146^ ^]^ The novel unit cell geometry was developed using an energy homogenization equation for the global displacement together with topology optimization. FEA and sampling analyses resulted in constructs with controllable and negative Poisson's ratios. In a recent computational approach, four auxetic unit cells were thoroughly tested for their mechanical performance as stent elements.^[^ ^144^ ^]^ The FEA models of the stents were subjected to crimp, crush, three‐point bending, and kink tests. Simulation results indicated that the designs with the best trade‐off between radial stiffness and bending flexibility were two hybrid geometries, namely, a chiral‐re‐entrant and a chiral‐star design.^[^ ^144^ ^]^ Using a novel unit cell design, based on a ring‐link configuration, Han and Lu presented an experimental stent with nonuniform Poisson's ratio that could effectively follow the curvature of arteries (Figure 18B(i)).^[^ ^141^ ^]^ Compared with stents showing a positive Poisson's ratio or a uniform negative Poisson's ratio across their entire length, the novel stent exhibited a bending behavior when expanded laterally using a balloon catheter (Figure 18B(ii)). Furthermore, the new design allowed a smooth transition between positive and negative Poisson's ratio regions, which translated into an equally smooth curvature transition from concave to convex regions, in simulations of stent deployment in a patient‐specific curved artery.

##### Ultraproperty Stents

Surjadi et al. presented a method for the fabrication of ultraproperty coronary stents.^[^ ^81^ ^]^ In this approach, a partial carbonization method was used to enhance the inherent high strength of the microlattices. The mechanical strength and energy absorption values of the pyrolized samples were found to be 100 times higher compared to their noncarbonated counterparts. Moreover, the ultrastrong lattices could deform to 50% strain without fracturing, which was almost double than the fracture strain of the controls, showcasing their extraordinary combination of high strength and ductility demonstrated by the partially carbonized microlattices. Furthermore, a better cell adhesion capacity was reported for the partially pyrolyzed lattices compared to the controls (Figure 18C(i)). This method was subsequently used to fabricate functionally coated core–shell coronary stents with a partially carbonized core (Figure 18C(ii–iii)).^[^ ^81^ ^]^

##### Origami‐Based Stents

A self‐deployable vascular stent made from a single foldable foil was presented by Kuribayashi et al.^[^ ^147^ ^]^ A shape‐memory alloy (Nitinol) foil was used as the origami “paper” while the pattern folds were created using negative photochemical etching. The hill and valley creases of the origami design were fabricated by etching each crease type on opposite sides of the stent (Figure 18D(i)). The designed creases allowed the stent to be folded into a smaller configuration, and to subsequently expand into its original size upon temperature increase (Figure 18D(ii)).

In general, using auxetic metamaterials in stent design has been reported to provide i) good fixation and radial expansion under compressive stress, ii) minimum recoil, and iii) minimum axial shortening upon delivery. Overall, there is an evident preference in the literature for auxetic geometries that feature a rotating node (chirals) or rigid shape (square) in their unit cell. However, there is no clear explanation for this trend. Although the majority of the revised works was focused on auxetic stent designs, the limited studies that implemented nonauxetic metamaterials revealed that they hold significant promise for future vascular stent research. Furthermore, the selected fabrication method may affect significantly the mechanical performance of the stents. In the reviewed studies, two fabrication approaches have been presented, i.e., single‐piece fabrication of 3D stents or fabrication of a 2D lattice, which was subsequently rolled into a tubular construct. In the latter approach, attention is needed to ensure that the rolling step does not introduce local stresses in the final stent. For instance, a final curing step could be added that would ensure relaxation of any rolling‐induced stresses.

#### 3.2.3 Vascular Grafts

##### Auxetic Grafts

**Figure 19:**
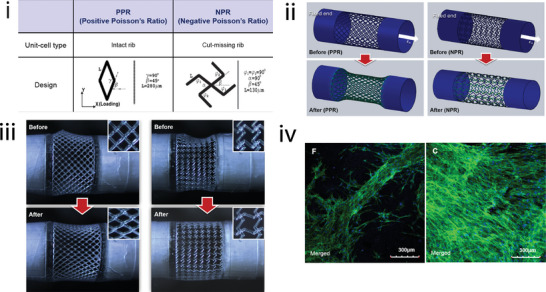
Mechanical metamaterials applications in vascular graft design. (i) Intact rib and missing‐rib unit cell designs for the positive Poisson's ratio and negative Poisson's ratio grafts, respectively. The independent geometric parameters are also presented in the schematic. (ii) FEA of the stress and deformation of tubular constructs composed of intact‐rib (left) or missing‐rib (right) architectures. (iii) Images of the tensile tests of the positive (left) and negative Poisson's ratio constructs (right). The radial expansion of the auxetic graft can be observed. (iv) Fluorescent images of hTMSCs stained actin filaments (green) and nuclei (blue) on the intact‐rib (right) and missing‐rib (right) constructs after 7 days of culture. Adapted under the terms of the CC‐BY license. Copyright 2016, PLoS One.^[^ ^137^ ^]^

One of the risks of implanting small diameter vascular grafts in vessels that experience high blood pressure, is a compliance mismatch between the graft and the native tissue, which causes problems in blood flow including turbulence or anastomotic intimal hyperplasia.^[^ ^148^ ^]^ This challenge was addressed by Lee et al. who proposed the use of auxetic grafts based on a missing‐rib unit cell geometry (**Figure** **19**(i)).^[^ ^137^ ^]^ Flat lattices were initially fabricated from poly(ethylene glycol) diacrylate (PEGDA) using a light‐based printing strategy and subsequently rolled into tubular constructs. Simulations and tensile tests on the rolled specimens indicated a negative Poisson's ratio for the missing‐rib stents for up to 19% strain (Figure 19(ii,iii)). The auxetic constructs could support a significantly higher cell attachment and proliferation for 7 days compared to nonauxetic, intact‐rib lattices (Figure 19(iv)). However, this result could be related to the unit cell geometry and pore size, rather than the auxeticity of the structure, since the in vitro experiments were conducted in static conditions. Another auxetic vascular graft design for compliance enhancement was suggested by Ahn et al.^[^ ^138^ ^]^ In this study, a three‐layer graft was fabricated in order to match the arterial wall architecture (tunica intima, tunic media, and tunica adventitia).^[^ ^149^ ^]^ The inner and outer layers of the graft were made by electrospun PCL fibers while the auxetic middle layer was designed using the re‐entrant honeycomb unit cell, by extrusion printing of PCL on a rotating rod system. The multilayered auxetic grafts exhibited Poisson's ratios between −0.75 and −0.533 under tensile loading and a 3.8 times better compliance compared to commercial grafts. HUVECs and VSMCs were seeded on the luminal and outer side of the graft, respectively. Both cell types showed high viability and proliferation rates for 10 days and formed layers on the inner and outer part of the graft. Furthermore, the contractile phenotype of VSMCs was successfully maintained in the coculture with HUVECs in the auxetic graft.^[^ ^138^ ^]^

Although the reviewed studies reported promising results for avoiding compliance mismatch between the graft and the surrounding tissue, the challenge of vascular tissue organization was not addressed. Native blood vessels consist of layers with highly aligned cells. More specifically, the innermost layer, the tunica intima consists of a monolayer of axially aligned endothelial cells,^[^ ^150^ ^]^ while the middle layer, the tunica media, consists of circumferentially aligned contractile smooth muscle cells.^[^ ^151^ ^]^ Thus, special attention should be given to the selected unit cell geometry to ensure that it does not compromise cell alignment and tissue organization. One possible approach for achieving this is the use of a supporting layer made of aligned electrospun fibers that could guide the cell organization as reported in other studies.^[^ ^152^, ^153^ ^]^

### 3.3 Skin

Skin is an organ that exhibits complex and out‐of‐plane deformations, owing to its elastic and soft nature. However, it is susceptible to defects that are commonly known as wounds.^[^ ^154^, ^155^ ^]^ Despite the ability of skin to self‐repair and restore its structural and functional integrity, wound care is crucial for preventing infection, accelerating the healing process and avoiding the formation of scar tissue. The necessity of wound care becomes more relevant for large and open burns and wounds.^[^ ^156^ ^]^ Additionally, the spontaneous repair of skin may be disrupted due to various pathologies.^[^ ^157^ ^]^ The most widespread wound healing method is wound coverage by dressings.

Wound dressings should closely match the mechanical behavior of native skin in order to enhance tissue repair and allow a range of motion as close to the physiological one as possible. However, skin exhibits complex mechanical properties, including an anisotropic, viscoelastic, and nonlinear stress–strain response.^[^ ^158^ ^]^ Furthermore, in vivo, skin is under tension, which varies based on the location, the age, and the patient.^[^ ^159^ ^]^ Hence, metamaterials could be promising candidates for the fabrication of dressings due to their superior mechanical properties.

#### 3.3.1 Ultraproperty Dressings

**Figure 20:**
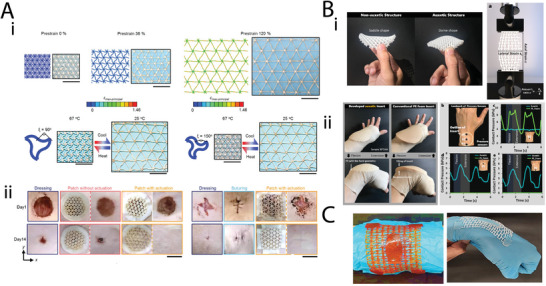
Mechanical metamaterials applications in skin tissue engineering. A) (i) Simulations and images of prestrained metalattices fabricated from a liquid crystal elastomer. The lattices could contract on demand upon temperature increase. Different unit cell angles resulted in distinct stress–strain, biaxial strain, and actuation stress values. (ii) Images from the progress of wound healing during in vivo experiments after 14 days. Reproduced with permission.^[^ ^103^ ^]^ Copyright 2021, John Wiley and Sons. B) (i) Representative images of the out‐of‐plane deformation of auxetic wound dressings and their lateral expansion upon uniaxial stretching. (ii) Comparison of applied pressure on a human wrist from a conventional foam‐based insert and the auxetic insert, recorded by three pressure sensors. Adapted with permission.^[^ ^104^ ^]^ Copyright 2022, John Wiley and Sons. C) Images of the applied wound dressing around curved body surfaces. Adapted under the terms of the CC‐BY license.^[^ ^105^ ^]^ Copyright 2023, MDPI.

Wu et al. presented a novel ultraproperty‐based liquid crystal elastomer that exhibited impressive biaxial actuation capabilities (i.e., biaxial strain of −53%) and a low actuation temperature of 46 °C (**Figure** **20**A(i)).^[^ ^103^ ^]^ Rationally designed, shrinkable patches made of the liquid crystal elastomer were tested in vivo and were found to accelerate skin regeneration while limiting scar and keloid formation in rats (Figure 20A(ii)).

#### 3.3.2 Auxetic Inserts

During the remodeling phase of wound healing, the formation of hypertrophic scars can negatively affect the mobility of joints and the function of surrounding tissues. The standard therapy approach for hypertrophic scars is pressure application, which accelerates tissue maturation and improves the appearance of the scar.^[^ ^160^ ^]^ Auxetic inserts have been studied for their superior pressure delivery abilities during out‐of‐plane deformation for the treatment of hypertrophic scars (Figure 20B(i)).^[^ ^104^ ^]^ Pressure evaluation experiments on a human wrist revealed that the double arrowhead insert outperformed its re‐entrant honeycomb counterpart and could provide the necessary pressure to the whole area it was covering during extension and flexion (Figure 20B(ii)).

#### 3.3.3 Auxetic Dressings

In a different approach, auxetic hydrogel wound dressings based on periodic elliptical and rectangular holes were proposed as a cost‐effective solution for wound healing (Figure 20C).^[^ ^105^ ^]^ The superior mechanical properties of the patches allow their application on a wide range of injury sites and their transparency can be combined with pH and glucose sensors for real‐time monitoring of the healing process. Furthermore, Liu and Zhang introduced novel auxetic lattices based on triangular and honeycomb grids with zigzag‐based edges.^[^ ^106^ ^]^ Simulation and experimental results indicated that the zigzag network designs could provide any Poisson's ratio in the range from −1 to 1 over a strain range of 0% up to ≈90%. The geometric parameters of the auxetic lattices could be tailored in order to closely match the Poisson's ratio as well as the stress–strain curve of cat skin.

Despite the promising results reported in the literature, the full potential of metamaterials in wound healing applications has not been thoroughly researched yet. More in vitro and in vivo studies are needed in order to screen different unit cell geometries and their effect on healing. Metamaterial‐based dressings could be further combined with antibacterial or conductive materials, to further accelerate the healing and remodeling processes.^[^ ^161^, ^162^ ^]^

### 3.4 Oesophagus and Trachea

#### 3.4.1 Auxetic Stents

In advanced stages of esophageal cancer, cancerous tumors develop on the inner layers of the esophageal lining and usually result in dysphagia and pain. Malignant esophageal obstruction may also occur because of extrinsic compression from adjacent lymph nodes or tumors arising in the mediastinal organs. One of the palliative treatment approaches is the use of soft, esophageal stents for the dilation of the narrowed, affected site, in order to allow more food intake, thus improving the patient's quality of life.^[^ ^163^ ^]^ Auxetic cylinders based on a rotating square design have been presented as promising esophageal stents.^[^ ^164^, ^165^, ^166^ ^]^ Owing to their negative Poisson's ratio, the stents can resist esophageal constriction due to the lateral compression on the stent's wall from the tumors.

**Figure 21:**
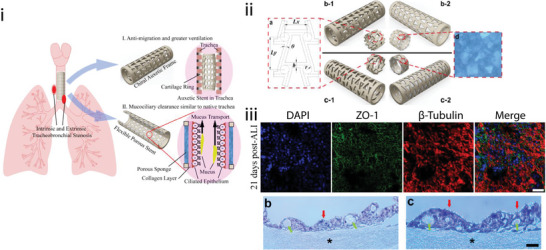
Mechanical metamaterials application for tracheal stents design. (i) Schematic of the auxetic tracheal stent concept. The chiral auxetic frame offers better fixation in the stenotic trachea, allowing a better ventilation, while the flexible porous sponge supports mucociliary clearance similar to the native trachea. (ii) The two configurations of the tracheal stent. The same tetrachiral unit cell was used either in a vertical (top) or a horizontal symmetry (bottom). The right inset illustrates the porous sponge layer added in both configurations. (iii) Top: immunostaining images and histology (bottom) of the formation of ciliated tracheal epithelium after 21 days in culture under air–liquid interface conditions. Reproduced with permission.^[^ ^169^ ^]^ Copyright 2021, Elsevier.

Tracheobronchial stenosis is a condition that can occur due to inflammation, malignancy, dynamic collapse or tuberculosis. Patients with significant stenosis may develop dyspnea, wheezing or stridor.^[^ ^167^ ^]^ In severe cases, stenosis can cause obstructive pneumonia or even suffocation. Similarly to the treatment of oesophageal obstruction, stenting is the principal approach used to manage the airway compression.^[^ ^168^ ^]^ An auxetic and porous stent based on a tetrachiral and anti‐tetrachiral hybrid unit cell was presented as a candidate for tracheal stenosis (**Figure** **21**(i,ii)).^[^ ^169^ ^]^ The auxetic stents demonstrated a near‐zero or negative Poisson's ratio for up to 50% strain, thus exhibiting a significantly larger ventilation diameter and increased antimigration ability compared to common stents. Moreover, the stents could support in vitro differentiation of a ciliated tracheal epithelium under air–liquid interface conditions (Figure 21(iii)).

### 3.5 Tendon and Ligament

#### 3.5.1 Ultraproperty Scaffolds

**Figure 22:**
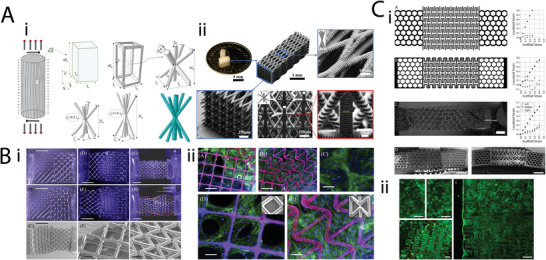
Mechanical metamaterials applications in tendon and ligament tissue engineering. A) (i) Different unit cell geometries for tendon and ligament created by changing the values of the independent geometric parameters. (ii) Images of the fabricated scaffolds at various magnifications. Reproduced with permission.^[^ ^23^ ^]^ Copyright 2022, Elsevier. B) (i) (Top) Images of a hybrid scaffold combining a region with a positive and a region with a negative Poisson's ratio. During tensile tests the conventional and auxetic responses can be identified. (Bottom) SEM images of the hybrid scaffold and close‐ups on the square grid (positive Poisson's ratio) and the re‐entrant honeycomb (negative Poisson's ratio). (ii) Actin (green) and nuclei (blue) staining of hMSCs growing on the two regions after 7 days in culture. Reproduced with permission.^[^ ^173^ ^]^ Copyright 2012, Elsevier. C) (i) Designs and images of a hybrid scaffold with two positive Poisson's ratio regions (honeycomb) and an auxetic region (re‐entrant honeycomb). (ii) Fibroblasts growing on the scaffold after 5 days in culture. Reproduced with permission.^[^ ^24^ ^]^ Copyright 2017, Elsevier.

Tendons are connective tissues that consist of tightly packed parallel collagen fiber bundles. Due to their hierarchical structure tendons possess unique biomechanical properties, such as viscoelasticity and high‐mechanical strength.^[^ ^170^ ^]^ Interestingly, healthy tendons of both animal and human origin have been reported to exhibit auxetic behavior. Since unhealthy tendons have been observed to lose their hierarchical organization, it has also been suggested that the Poisson's ratio may be used as an indicator of tendon health.^[^ ^4^ ^]^ Hence, to effectively mimic the mechanical response of healthy tendon, auxetic metamaterials may be considered. Karathanasopoulos and Al‐Ketan presented an innovative unit cell design based on a body‐centered cuboid geometry to address the lack of conventional materials capable of recapitulating the mechanical response of tendons (**Figure** **22**A).^[^ ^23^ ^]^ Six distinct unit cells were designed by increasing the relative edge difference of the beams. Specimens based on the aforementioned unit cells were fabricated specifically for compression and shear tests. Simulation and experimental results showed a substantial normal stiffness along their primal loading direction that was up to 18 times greater than their shear resistance, closely matching the response of native tendons.

#### 3.5.2 Hybrid Scaffolds

Another extraordinary feature of tendons is their ability to connect to the much stiffer bone tissue, at the enthesis, along a relatively short transitional length.^[^ ^171^ ^]^ Engineering the soft–hard interface of the osteotendinous junction is a challenge that could potentially be addressed by tuning the design parameters of the unit cell.^[^ ^172^ ^]^ One suggestion to achieve this transition is to combine areas of negative and zero or positive Poisson's ratio on the same construct.^[^ ^24^, ^173^ ^]^ In a different study, scaffolds were fabricated by combining auxetic re‐entrant honeycomb lattices with nonauxetic square grids. Simulation and experimental results demonstrated that the two regions of the scaffolds exhibited opposite Poisson's ratios and could support growth and proliferation of hMSCs for 7 days (Figure 22B).^[^ ^173^ ^]^ A hybrid scaffold combining normal and re‐entrant lattices was presented in a study by Warner et al.^[^ ^24^ ^]^ Actuation experiments revealed that the mechanical response of the hybrid scaffold to tensile loading was similar to the one simulated using FEA. Moreover, in vitro experiments showed that the hybrid constructs could support adhesion and growth of fibroblasts and myoblasts for up to 5 days (Figure 22C).

### 3.6 Neural Tissue

The nervous system is a complex network that controls a host of body functions, including cognition and individual cell processes.^[^ ^174^ ^]^ Nerve regeneration is an elaborate process that depends on the site and the extent of the injury. For instance, in the peripheral nervous system, nerves can regenerate on their own if the injuries are not large. Conversely, in the central nervous system, axon regeneration is limited.^[^ ^175^ ^]^ A promising approach for nerve regeneration is the differentiation from pluripotent stem cells (PSCs), which has been used in disease modeling and drug screening for the treatment of neurological diseases. A key player for the differentiation process, is the biophysical signaling from the scaffolds to PSCs. Neural lineage commitment is very sensitive to the elastic modulus of the substrates, and low elastic moduli (<10 kPa) have been reported to promote neuronal differentiation.^[^ ^176^, ^177^ ^]^ Nevertheless, the impact of Poisson's ratio on stem cell fate remains less explored.

#### 3.6.1 Auxetic Scaffolds

**Figure 23:**
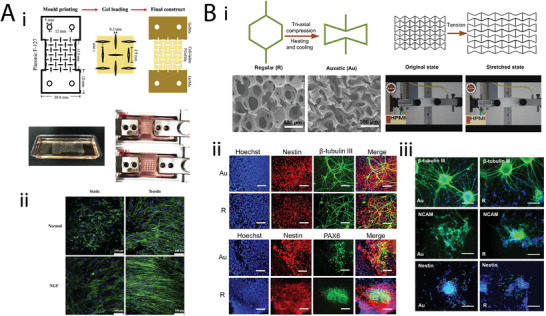
Mechanical metamaterials applications in neural tissue engineering. A) (i) Schematic representation of the fabrication of rotating square scaffolds, and photos of the as‐fabricated scaffold as well as at 20% strain. (ii) Fluorescent images of Schwann cells cultured in the scaffolds under static or dynamic tensile loading. Reproduced with permission.^[^ ^178^ ^]^ Copyright 2020, Elsevier. B) (i) Schematic illustration of the re‐entrant honeycomb‐like unit cells obtained from triaxially compressed foams (top). SEM imaging and tensile tests of the auxetic foams (bottom). Enhanced differentiation of human iPSK3 cells (ii) and mouse ESCs (iii) in auxetic, triaxially compressed scaffolds. Reproduced with permission.^[^ ^179^ ^]^ Copyright 2017, Elsevier.

To investigate this effect, Chen et al. presented auxetic, Schwann cell‐laden hydrogels, based on perforated sheets that acted as a rotating square system (**Figure** **23**A).^[^ ^178^ ^]^ The scaffolds were stimulated during culture by applying a 20% tensile strain at a frequency of 0.48 Hz. Cells cultured under cyclic tension showed enhanced alignment and higher expression of differentiation markers, compared to cells in static culture. It should, however, be noted that the auxetic effect of the scaffolds was not fully investigated, due to the lack of nonauxetic controls.

In a different study, Yan et al. created auxetic polyurethane foams with re‐entrant hexagonal profiles to enhance neural regeneration.^[^ ^179^ ^]^ The structures were created with different moduli (10–100 kPa) and Poisson's ratio values (−0.45 to 0) and were compared to traditional nonauxetic foams. in vitro experiments with mouse embryonic and human‐induced pluripotent stem cells revealed that the auxetic scaffolds induced a higher neuronal marker expression compared to the nonauxetic controls (Figure 23B).

It is worth mentioning that the electrical properties of nerve cells were not considered in the abovementioned studies. Since electrical stimulation has been shown to enhance the nerve regeneration process,^[^ ^180^, ^181^ ^]^ it would be interesting to combine conductive materials (e.g., polypyrrole or polyaniline^[^ ^182^ ^]^) with metamaterial geometries in order to study the combined effect of electrical and mechanical actuation.

### 3.7 Vertebrae and Intervertebral Discs

A herniated lumbar disc occurs when the disc material (nucleus pulposus or annulus fibrosus) is displaced beyond the intervertebral disc space.^[^ ^183^ ^]^ The herniation may place pressure on nearby nerves or the spinal cord and, in severe cases, may need to be treated with a disc prosthesis.^[^ ^184^ ^]^ Using a prosthesis with auxetic properties could prevent implant bulging, thus minimizing any adverse effects on the nerves and the mechanical behavior of the spine.

#### 3.7.1 Auxetic Implants

An early theoretical analysis and design of a disc replacement was presented by Martz et al. The implant was designed initially based on a reversed honeycomb unit cell and was found to possess a Poisson's ratio in the −0.9 to −0.6 range, based on FEA.^[^ ^185^ ^]^ In a more recent work, a novel auxetic implant with a bucklicrystal‐like design has been presented as a potential intervertebral disc replacement, in cases of severe lumbar disc herniation. Quasi‐static and cyclic compression tests showed that the bucklicrystal scaffolds were stiffer and possessed negative Poisson's ratios ranging from −0.52 to −0.32 at strains of 10% to 50%, compared to the ratios of conventional implants that were between 0.19 and 0.47 for similar strains. Simulation results indicated that the bucklicrystal‐based implant could re‐establish native spine motion and alleviate lumbar disc herniation. in vitro experiments confirmed the biocompatibility of the auxetic implants and in vivo implantation in rabbits revealed that the specimens could withstand the spinal load while maintaining the natural motion of the spine.^[^ ^186^ ^]^

#### 3.7.2 Kirigami and Origami Implants

**Figure 24:**
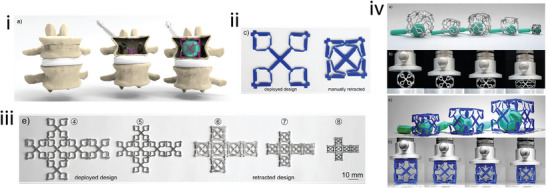
Mechanical metamaterials application for vertebral implant design. (i) Schematic of the application of a deployable vertebral metaimplant to fill the vertebral cavity. (ii) The unit cell of the metaimplants before (left) and after retraction (right). (iii) A large and a small implant prototype before and after retraction. (iv) Different implant 3D configurations after deployment using a balloon and during retraction. Adapted under the terms of the CC‐BY license.^[^ ^22^ ^]^ Copyright 2020, Elsevier Ltd.

A novel concept for the fabrication of deployable metaimplants for the treatment of vertebral compression fractures was presented by Bobbert et al. (**Figure** **24**).^[^ ^22^ ^]^ The implants were designed based on the kirigami and origami principles, starting from a flat configuration, and, upon request, could be deployed to a new 3D stable state to precisely fill the vertebral cavity.

### 3.8 Effect of Mechanical Metamaterials on the Cellular Level

The effective elastic modulus of scaffolds and the Young's modulus of their constituent biomaterials have been reported to affect cell response through mechanotransduction pathways in various applications. Although the elastic modulus effect on the cellular level has been widely studied, the effect of Poisson's ratio has not yet been investigated and understood in depth. A possible reason for this gap is the challenge to decouple the effects of Poisson's ratio from those of other scaffold parameters, such as the porosity, pore size, surface area, and elastic modulus. Furthermore, investigating the auxetic effect at the cellular level requires the fabrication of complex constructs with dimensions relevant to those of individual cells, which is a significant challenge. Nevertheless, a few promising attempts have been presented in the literature, demonstrating impressive results.

#### 3.8.1 Auxetic Structures

**Figure 25:**
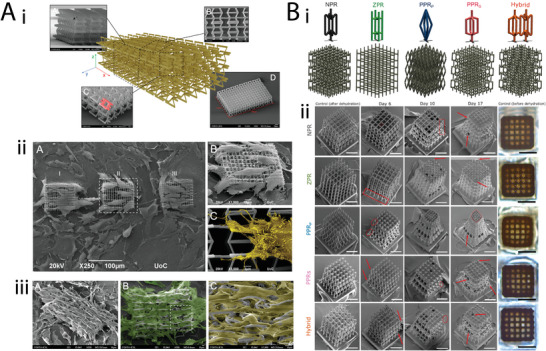
Mechanical metamaterials applications at the cellular level. A) (i) Design and fabrication of auxetic microlattices based on a re‐entrant honeycomb unit cell. (ii, iii) 3D, auxetic microlattices with attached mouse fibroblasts. The microscopic scaffolds can be seen to deform under the forces applied to them by the fibroblasts. Reproduced with permission.^[^ ^189^ ^]^ Copyright 2020, John Wiley and Sons. B) (i) Unit cell and scaffold designs for the fabrication of constructs with a range of Poisson's ratio values. (ii) SEM images of the scaffolds after dehydration and on days 6, 10, and 17 of mouse preosteoblasts culture. Different deformation modes can be observed depending on the Poisson's ratio value. Reproduced with permission.^[^ ^192^ ^]^ Copyright 2024, Elsevier.

Auxetic structures with tunable Poisson's ratio for the investigation of cellular behavior were fabricated by Zhang et al. Embryonic fibroblasts were seeded on the microlattices and could apply forces on the lattice nodes that resulted in a local deformation. It was also observed that auxetic lattices could deform much more due to cellular migration, when compared to their nonauxetic counterparts.^[^ ^187^ ^]^ In a different approach, deep reactive ion etching was used to fabricate auxetic cantilevers and bridges of a few micrometers. The resonant frequencies of both structures and designs were investigated through FEA. A resonant sensor for monitoring cell growth was also investigated by comparing the resonant frequencies of the auxetic microlattices in the absence and presence of a single eukaryotic cell attached on them. In vitro experiments with hMSCs showed that the cells were interacting with the microlattices by migrating on top of them.^[^ ^188^ ^]^ Microscopic auxetic scaffolds fabricated using multiphoton lithography and 3D re‐entrant honeycomb unit cells were found to promote mouse fibroblast attachment and penetration. The unit cell edges were observed to deform and buckle due to the tensile forces applied by the fibroblast filopodia (**Figure** **25**A).^[^ ^189^ ^]^ In a recent study, metamaterial microscaffolds were fabricated using two photon‐polymerization, with different Poisson's ratios while exhibiting similar values of porosity, pore size, strut diameter, and effective elastic modulus (Figure 25B(i)).^[^ ^190^ ^]^ Mouse preosteoblasts could attach and populate scaffolds with both negative and positive Poisson's ratios, while osteogenic markers were expressed in all conditions. Matrix mineralization was similar in all conditions; however, the proliferation of the preosteoblasts on the metamaterials with negative Poisson's ratio was lower than that observed for the conventional geometries. Interestingly, cell adhesion resulted in different scaffold deformations depending on the value of their Poisson's ratios (Figure 25B(ii)).^[^ ^190^ ^]^ Aerogels with negative Poisson's ratios have also been used to study the auxetic effect on mouse bone marrow MSCs.^[^ ^191^ ^]^ Interestingly, it was reported that the negative Poisson's ratio significantly affected cell proliferation and enhanced chondrogenic differentiation, compared to aerogels with positive or zero Poisson's ratio values.

It has been, therefore, shown that the Poisson's ratio has a distinct effect on cell behavior and could be a useful tool to affect in vitro proliferation, adhesion, and differentiation. However, more research is needed to investigate how auxeticity could affect different cell types. Moreover, other types of metamaterials, such as ultraproperty materials, could also be researched more in depth for their influence on the fate of individual cells. Finally, it would be worth to further investigate the quality and quantity of the formed matrix upon cellular interaction with mechanical metamaterials.

## 4 Future Prospects and Challenges

In this work, we have summarized mechanical metamaterial classes with a special focus on the unit cell designs and their essential geometric parameters. We have discussed the emergence of AI‐aided metamaterial design and its promising potential for tissue regeneration. Finally, we have presented the current state‐of‐the‐art of metastructures to guide growth, regeneration, and new tissue formation.

Despite the promising potential of mechanical metamaterials for tissue engineering applications, there are limitations in the current approaches that need to be addressed. In most of the reviewed body of literature, the mechanical performance of the fabricated metamaterial constructs was evaluated by FEA simulations or quasi‐static mechanical tests. Due to the complexity of the metamaterial geometries and the variety of reviewed applications, mechanical characterization with conventional protocols was not always sufficient, and novel testing methods had to be introduced.^[^ ^112^, ^137^ ^]^ Therefore, in order to allow reliable validation of results, new test standards that address metamaterial responses could be proposed.^[^ ^14^ ^]^

Even when the loading direction of the investigated construct complies with typical uniaxial compressive or tensile tests, a quasi‐static approach is often used for mechanical characterization.^[^ ^111^, ^113^ ^]^ However, the mechanical loading experienced by native tissues is mostly of dynamic nature. Therefore, more studies are needed to investigate the cyclic response of metamaterials and explore how it could be matched to the dynamic response of target tissues. In addition, dynamic loading has been observed to instruct cell differentiation and tissue maturation.^[^ ^193^, ^194^ ^]^ For instance, in cartilage engineering, the loading frequency for cyclic tests is often selected to resemble human walking (i.e., 1 Hz).^[^ ^195^ ^]^ Although a few of the reviewed studies have considered dynamic loading during cell culture,^[^ ^121^, ^178^ ^]^ it is still unclear how metamaterials could affect cell response under cyclic mechanical stimulation. Since the extraordinary mechanical responses of metamaterials are exhibited under loading, dynamic culture conditions using tensile/compressive bioreactors would aid in unraveling any metamaterial effects on cell response.^[^ ^196^ ^]^ Furthermore, metamaterials could be fabricated using “active” constituent materials (e.g., magnetically, acoustically or electrically responsive) to permit remote mechanical stimulation in vitro. Control conditions require cautious selection to ensure that any differences in cell behavior can be reliably attributed to the metamaterial effect.

While the properties of the constituent material are not discussed in this review, they are of importance both for the mechanical integrity of the final structures and, more specifically, for their application in tissue engineering. Adopting a term thoroughly discussed by Zadpoor,^[^ ^197^ ^]^ the design of “meta‐biomaterials” includes an additional level of complexity that addresses the biocompatibility, toxicity and, when closer to clinical translation, regulatory aspects. Those properties depend majorly on the bulk material used, however, the metamaterial structure itself could affect them. For instance, the porosity and total free surface of the scaffolds or implants is directly related to their degradation rate. Another point of consideration is the mechanical performance of metamaterials during the degradation process. For example, if the degradation of thin struts results in a decrease of their thickness, the Poisson's ratio of the entire lattice is expected to change, since thickness is among the independent geometrical parameters of many metamaterials. Therefore, meticulous studies of the progressive mechanical and chemical changes of the implants, together with the maturation of the surrounding tissues, could provide a valuable insight. From the mechanical performance scope, the bulk properties of the constituent material are far from irrelevant. The elastic properties of the individual unit cell features should be such, as to allow for the elastic deformation of the entire construct up to the desired strain range. Considering that native tissues are subject to a host of loading modalities with various strain ranges and frequencies, the metamaterial‐based scaffolds/implants should also be able to support dynamic loading. Another level of complexity is introduced when the long‐term, dynamic, mechanical performance of those implants is considered. Further research is required to investigate how to couple tissue integration with implant/scaffold degradation under constant dynamic loading. Ideally, a smooth transition between the scaffold‐supported to the tissue‐dominated response should be achieved. While there is no clear guideline for the ideal property range of constituent materials, an overview of the materials already used in tissue engineering applications reveals a clear trend for soft, elastic polymers, hydrogels, and soft metals (Table 2).

Similarly to conventional biomaterials, the tissue growth and maturation around and inside the metamaterial‐based scaffolds or implants in vivo is another point requiring investigation. Going beyond the obvious routes to avoid a foreign body response, such as the selection of an appropriate constituent material, the scaffold/implant decoration with releasing anti‐inflammatory cytokines and matching the mechanical properties of the surrounding tissues, the extraordinary properties of metamaterials could be regarded as a platform to investigate improved biointegration or even limitation of foreign‐body response. For instance, the biomechanical effect on immunomodulation could be considered as another layer of design considerations. Hence, different unit cells could be studied in vitro for their effect on macrophage polarization into the M2 (alternatively activated) lineage during dynamic loading.^[^ ^198^ ^]^ Therefore, a novelclass of immunoinformed meta‐biomaterials could be created, which would combine extraordinary properties with geometry‐dependent immunomodulation.

In addition, limited knowledge is present in the current literature over the mechanical effect of metamaterials on cell fate. More research is needed on the cellular level, to understand how cell proliferation and differentiation can be affected by metamaterial responses. As a first step toward this direction, research could focus on the use of scaffolds and constructs of comparable size to those of cells, and subsequently compare the results to the effect of larger constructs. However, as mentioned already, if the scaffolds are not subjected to mechanical stimulation during cell culture the auxetic effect could be reduced to a mere pore‐size or geometry effect. Although a few studies have attempted to explore the effect of auxeticity at the cellular level,^[^ ^187^, ^188^, ^189^, ^190^ ^]^ most in vitro experiments use 2D lattices. However, the mechanical response of a 3D metamaterial construct under loading could be significantly different from the response of a 2D lattice, thus having a distinct effect on cells seeded in it. Further research is, therefore, required to decipher the differences of the biological effects between 2D and 3D structures.

Depending on the unit cell geometry, different fabrication methods may or may not be considered. For instance, auxetic lattices or kirigami planes could be fabricated by both layer‐by‐layer and volumetric printing techniques. When extrusion printing, such as FDM and DIW, is used, the design and planning of the printing path could require thorough optimization in order to minimize the number of stops and restarts, thus minimizing the fabrication time, while ensuring a higher structural fidelity of the final construct.^[^ ^199^ ^]^ On the other hand, more complex metamaterials, such as bucklicrystals, might be more challenging to fabricate using a layer‐by‐layer approach, regardless of the printing path followed. For complex 3D unit cell geometries, volumetric fabrication methods could provide a faster and more robust result.^[^ ^200^ ^]^ Furthermore, the selected fabrication method could further affect the design parameters of the unit cell. For example, extrusion‐based methods will have a direct effect on the strut thickness of an auxetic lattice, as it will be a multiple of the inner diameter of the nozzle. Since the thickness is an independent geometric parameter, the fabrication method could impose additional constraints to the value range of the independent parameters.

As presented above, AI‐driven inverse design and unit cell geometry prediction is expected to have a substantial impact on novel metamaterial research, especially for tissue engineering. The possibility to obtain performance‐oriented structures by providing the desired response of the final construct to an AI model, could facilitate the design and fabrication of patient‐specific implants.^[^ ^201^ ^]^ Furthermore, AI models could be expanded to include other parameters that are relevant to biological applications. For instance, the biodegradation rate of the implant could be tuned by controlling its porosity, volume and total area.^[^ ^202^ ^]^ Nevertheless, there are challenges to be addressed in the development of accurate AI models. In order to accelerate the progress of AI‐generated metamaterials for tissue engineering and make them accessible to the scientific community, computation costs for extensive simulations should be minimized. Additionally, the AI models will need to be calibrated using reliable and robust databases, to ensure that the obtained results are indeed performance oriented and could be safely transferred to clinical applications.

Moreover, the effect of the unit cell geometry on the final mechanical properties of the metamaterial could be further exploited by introducing functional gradients or hybrid designs. Continuous variations of the geometric parameters could lead to graded distributions of material properties, resulting in superior mechanical performance.^[^ ^203^, ^204^ ^]^ The combination of discrete unit cell geometries in a single hybrid construct has been reported to provide a similar effect. Although this concept has received significant research interest, its implementation in tissue engineering is still in its infancy.^[^ ^112^, ^114^ ^]^ Metamaterial scaffolds could be developed by combining unit cells with graded parameters (e.g., increasing re‐entrant angle) and used in tissue engineering applications where a transition in mechanical response is desired, such as in soft–hard tissue interfaces^[^ ^172^ ^]^ or in sites where complex stress distributions are observed (e.g., the hip stem).^[^ ^205^ ^]^ However, it should be noted that combining different geometries in a single lattice may be challenging from a design perspective, especially at the border regions (i.e., the lattice points where the two different designs meet).

Although mechanical metamaterials have been used for the regeneration of various tissues, there still remains plenty of space for exploration. One field that could significantly benefit from the use of expanding constructs is that of pediatric implants. A child's natural growth may hamper the function of medical implants, as they are typically fabricated at fixed sizes. Using metamaterial geometries that can grow in size when their site of attachment grows or changes due to the child's development, could have a great impact on the health and quality of life of pediatric patients, by decreasing the number of implant replacement surgeries a child must endure.^[^ ^206^ ^]^ Metamaterials with multiple stable states could be considered for such applications, to provide long‐term geometric fidelity. Furthermore, the constituent materials should be chosen to have suitable degradation profiles, to allow for reliable mechanical deformations over the course of years.

## 5 Conclusion

Rationally designed metamaterials are gaining significant research momentum due to their extraordinary properties. Their hierarchical similarity to native tissues has led to an increasing interest in the use of mechanical metamaterials in tissue engineering. Nevertheless, the design of metamaterial constructs may be a challenging task that delays their wider spread within the biomedical field. We have, thus, presented a comprehensive overview of different mechanical metamaterials, focusing on the geometric parameters that are essential to their design and fabrication. The emergent field of AI‐assisted metamaterial design has also been introduced, in an effort to give a brief insight on the future of rationally designed materials. Furthermore, we have presented a thorough outline of current metamaterial applications in tissue engineering, in order to identify potential gaps and future research directions. Additionally, we have discussed the major challenges in the current research state and suggested future research steps to overcome them. We believe that our work can be used as a guide for the design of metamaterial‐based constructs and may inspire biomedical researchers to explore their promising potential. Novel mechanical metamaterial designs and applications are expected to lead to significant tissue engineering advancements and could likely become the preferred biomaterial geometries for the fabrication of scaffolds and biomedical devices.

## Conflict of Interest

The authors declare no conflict of interest.
